# Self-Trapped-Exciton Radiative Recombination in β–Ga_2_O_3_: Impact of Two Concurrent Nonradiative Auger Processes

**DOI:** 10.1021/acsaelm.4c02099

**Published:** 2025-02-17

**Authors:** Vytautas Grivickas, Patrik Ščajev, Saulius Miasojedovas, Lars Voss, Paulius Grivickas

**Affiliations:** † Institute of Photonics and Nanotechnology, Faculty of Physics, Vilnius University, Saulėtekio ave. 3, 10257 Vilnius, Lithuania; ‡ Materials Engineering Division, Lawrence Livermore National Laboratory, 7000 East Ave, Livermore, California 94550, United States; § Lawrence Livermore National Laboratory, 7000 East Ave, Livermore, California 94550, United States

**Keywords:** ultrawide bandgap electronic materials, anisotropic radiative emission, nonradiative lifetime, self-trapped polaron, Auger recombination mechanism

## Abstract

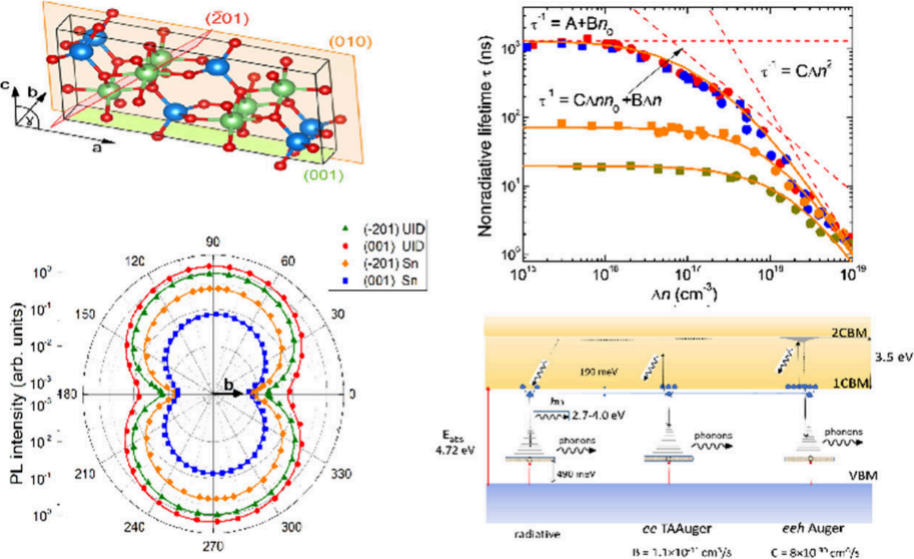

The peculiarities of radiative and nonradiative processes associated with self-trapped intrinsic eXcitons in the excited β-Ga_2_O_3_ crystals are studied via time-resolved techniques of induced absorption, transient grating, and photoluminescence (PL) at room temperature. The excitation above the bandgap is produced by laser pulses with linear light polarization parallel and orthogonal in the (−201) and (001) planes. We elucidate that the nonradiative recombination rate occurring in the eXciton prevails over its radiative emission rate in a wide range of free carrier concentration composed of excited and equilibrium electrons. Hence, the nonradiative recombination has no effect on the strong anisotropy and the shape of the eXciton emission band. However, we find out that the conventional ABC model of electron effective lifetime is insufficient for explanation of the excitation dependences. Inclusion of two nonradiative Auger mechanisms in a modified ABC formula provides excellent agreement of these dependences. We conclude that the trap-assisted Auger process is in proportion to the free electron density with coefficient B = 1.1 × 10^–11^ cm^3^/s and appears at low/intermediate excitation, while the triple-particle Auger process is in proportion to Δ*n*^2^ with coefficient C = 8 × 10^–30^ cm^6^/s and appears at high excitation conditions. The transition between two Auger mechanisms is accompanied by a rise of the eXciton diffusivity in preferred crystallographic directions where the radiative PL intensity is maximal. The diffusion length *L*_D_ in these directions can reach values ∼300 nm, but, at high excitations, *L*_D_ becomes limited by Auger lifetimes. These findings pave the way for the implementation of self-trapped eXcitons into specific optoelectronic devices.

## Introduction

Ga_2_O_3_ is an emerging ultrawide bandgap material with distinct anisotropic properties determined by its uncommon crystallographic structure. This compound’s crystals are of significant interest for next-generation power and optoelectronic devices across various practical applications. The most stable phase is the monoclinic β–polymorph.^1^ Unlike other wide-bandgap semiconductors such as AlN, cubic BN, and diamond, large β–Ga_2_O_3_ wafers can be produced at relatively low temperatures without requiring extreme pressures.^2^ Over the past decade, unintentionally doped (UID) and doped β–Ga_2_O_3_ commercial substrates of high structural quality and low level of Ga or O vacancies became available. It was shown that such substrates allow the *n*- or *p*-type conductivity epi-layers to be grown in a wide doping range.^3^ Additionally, chemical hydrogen treatment of Ga_2_O_3_ has been demonstrated to effectively induce shallow donors or transform them into shallow acceptors.^4^

The β–Ga_2_O_3_ polymorph is monoclinic, with a unit cell belonging to space group *C*2/*m*. This structure contains 20 atoms and features a notably long crystallographic base axis of *a* = 12.21 Å, a short base axis of *b* = 3.04 Å, and an intermediate axis of *c* = 5.80 Å. The monoclinic angle between the *a* and *c* directions is 103.7°. The crystal contains two distinct Ga^3+^ ion sites and three O^2–^ ion sites, with varying distances between them. Its structure is composed of alternating tetrahedral and octahedral arrangements, resulting in two easy cleavage planes. As a result, 2D flakes can be exfoliated, making them suitable for miniature applications.^5,6^ The crystal anisotropy leads to many Ga_2_O_3_ features where the bandgap edge anisotropy is the most-studied one.^7−9^ It was shown that the conduction band minimum (CBM) is constituted mainly by isotropic Ga 4s orbitals, while the valence band maximum (VBM) is formed from an oxygen anisotropic sublattice by 2p orbitals. The direct optical transition allowed for oriented dipoles. The value of the lowest direct bandgap *E*_G_ increases from 4.5 to 4.9 eV on Γ points for incident polarized light *E*_G,E//***c***_ < *E*_G,E//***a***_ < *E*_G,E//***b***_.^9−11^ The indirect band is less than 0.1 eV below the lowest direct transition, and its contribution is weak because of flatness of the VBM.^10^

The bandgap absorption and radiative emission properties of β–Ga_2_O_3_ differ significantly from those of conventional wide-gap semiconductors. Even at T → 0, the β–Ga_2_O_3_ crystals do not show sharp Wannier-Mott exciton involvement at the band edge in the absorption and emission. This phenomenon is attributed to the rapid formation of hole self-trapping as small polarons in a distorted dielectric environment. The Coulomb attraction of low mass free electron to polaron has been confirmed by many experiments.^12,13^ The excitonic origin of this behavior has been resolved using photocurrent excitation spectroscopy, even at steady states. These studies observed spectral shifts of the excitonic generation band under an applied electric field in Schottky diodes.^14,15^ According to these authors, we use the *eXciton* name in the following text to signify all specific properties of this exciton. The density functional calculations show that hole-polaron trapping reaches energy of about 0.49 eV, which is the largest for all classes of relevant metal oxides.^16,17^ The hole takes up position around either a single O atom or shares it on two closest O atoms.^18^ A few experiments performed by above band gap excitation revealed that the hole-polaron in β–Ga_2_O_3_ at 300 K is created in sub-femtosecond times.^19,20^ Once the hole-polaron becomes active, it attracts a low mass free electron with binding energy of ∼0.19 eV, and the effective eXciton Bohr radius is assumed to be 0.7 nm.^14^ It was shown that the thermal broadening in eXciton band (∼120 meV) correlates to the shrinkage of the bandgap ∼250 meV in the extended temperature range of 10–350 K. Both dependences can be simulated using a strong electron–lattice coupling (ELC) effect. Using an optical phonon energy of 31 meV, modeling suggests a large Huang–Rhys factor S ≈ 9.^18^ When β–Ga_2_O_3_ samples are doped with deep acceptors (e.g., Mg, Ca, Fe, Zn) or subjected to irradiation/implantation,^21−25^ hole self-trapping occurs at acceptors or at V_Ga_ or V_O_ vacancies. The appearance of donor–acceptor pair (DAP) polarized emission bands competes with the intrinsic eXciton band.^11,26^

To the best of our knowledge, nonradiative recombination of electrons to hole-polarons in β–Ga_2_O_3_ has not been systematically addressed. Previous studies have detected nonlinear carrier relaxation^19,26,27^ but lacked detailed analyses of transient decay dynamics. In the current work, we provide meaningful investigation of these intriguing characteristics by combining experiments including time-resolved techniques of induced differential absorption (Δα) and transient grating (TG) by diffraction efficiency (DE) with an optical probing. We also perform time-resolved studies of the eXciton radiative band and measure its radiative internal quantum efficiency (IQE). All investigations are made at 300 K in β–Ga_2_O_3_ samples of unintentional (UID) and *n*-type (Sn) doping at low-compensation. Calibrated, linearly polarized light above the absorption edge was used for the excitation. Our analysis reveals that nonradiative Auger processes dominate effective lifetimes across a broad excitation range. Recognition of these mechanisms uncovers novel photophysical insights and highlights the role of self-trapped excitons in metal oxide semiconductors, paving the way for enhanced functional properties for optoelectronic applications.^16^

## Samples and Their Optical Characterization

The study was carried out on bulk, epi-ready, β-Ga_2_O_3_ substrates synthesized by the EFG method and purchased from Novel Crystal Technology *Ltd.* The UID (−201) orientation wafer was of 2 in. in diameter, the rest of the samples were 10 × 15 mm^2^ in size and had both polished face surfaces. The sample parameters are indicated in Table 1. We performed Raman scattering measurements at confocal 532 nm continuous wave (CW) excitation. The polar plots of Raman lines were used to determine the orientation of different samples in (−201) and (001) planes,^28,29^ see Supporting Information S1. The *b*-axis exists at 0° polarization angle in both planes, e.g., (001) and (−201). These directions correspond to a higher allowed bandgap in β–Ga_2_O_3_, thus to a relatively low absorption for the 263–266 nm laser light wavelengths as shown below. In the following text, a simplified abbreviation is used: (//) – for light polarization angle 0°, and (⊥) – for light polarization with angle 90° to the orthogonal direction. The (⊥) direction corresponds to lower bandgaps and to higher absorption for 263–266 nm wavelengths, that is, to the E//(***a***) direction on the plane (001) and to the [102] direction on the (−201) plane.^9,30^ Before presenting the main results of carrier lifetimes, let us show basic optical parameters of the investigated samples that allows one to compare them to typical data from literature.

**Table 1:** Parameters of the investigated β-Ga_2_O_3_ samples

Orientation doping	Thickness (mm)	*n*_0_ (cm^–3^) = *N*_D_ – *N*_A_	τ_rad_ (μs)	τ_nr_ (μs) LL-exc.	τ_eff_ (ns) LL-exc.	IQE (%) LL-exc.
(−201) UID	0.62	6 ± 3 × 10^16^	8 ± 1.5	1.4	1100–1200	14
(001) UID	0.61	6 ± 2 × 10^16^	8.2 ± 1.5	1.7	1400	17
(−201) Sn-doped	0.65	1.25 ± 0.2 × 10^18^	2.6 ± 0.4	0.082	80	2.7
(001) Sn-doped	0.63	4.6 ± 0.2 × 10^18^	1.8 ± 0.25	0.018	12–24	0.9

### Absorption

**Figure 1:**
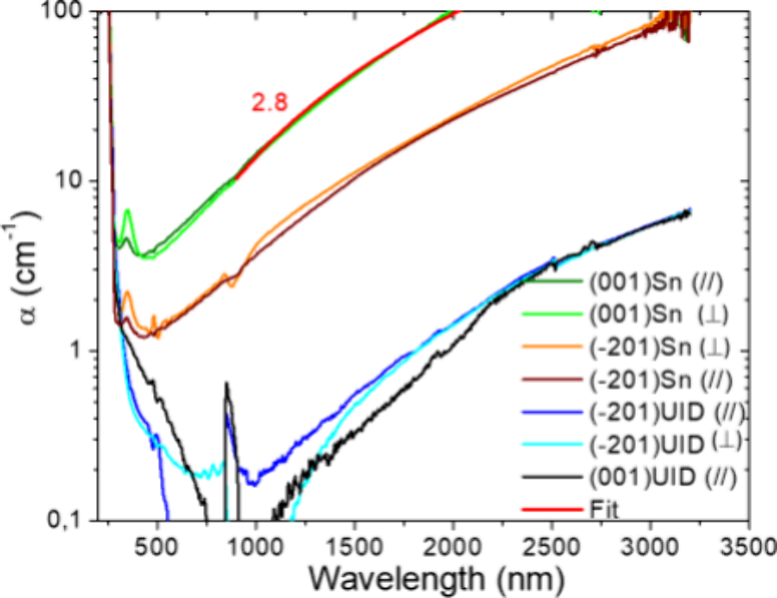
Absorption spectra in β-Ga_2_O_3_ substrates at two polarizations for determination of equilibrium free electron concentration: *n*_0_ = *N*_D_ – *N*_A_ (cm^–3^), see Table 1. The fitting function in the NIR region is α = σ_e_λ^2.8^ shown by the red line on the most Sn-doped (001) sample. The artifacts around 520 and 850 nm are due to spectrometer rearrangement.

The equilibrium electron concentration in the samples was obtained using optical transmission measurements in the visible-near-infrared (NIR) range, as shown in Figure 1. At wavelengths λ ≥ 1000 nm, the absorption increases in proportion to the free equilibrium electron density. Under a small acceptor compensation with *n*_0_ = *N*_D_ – *N*_A_ (cm^–3^), it can be explained by the Drude-type process due to intraconduction band optical transitions. The absorption is independent of light polarization due to the contribution from assisting phonons.^31^ Such a feature is pointed out in Figure 1 by a coincidence of two measured curves for (//) and (⊥) probe light polarization. The absorption dependence on wavelength is expected to be a function α ∼ λ^p^.^32^ We found an appropriate fit using α = σ_e_ × λ^2.8^ (*N*_D_ – *N*_A_) as shown by red lines in Figure 1. The power exponent gets into the error range of previously reported *p* = 3 ± 0.35 in doped and photoexcited β-Ga_2_O_3_.^33^ From wavelength proportionality, and in agreement to vendor’s reported doping in the most Sn-doped β-Ga_2_O_3_ (001) sample, e.g., *n*_0_ = 4.6 × 10^18^ cm^–3^ at λ = 1.55 μm, we extracted the value of free-electron cross-section for single electron σ_e_ = (1.0 ± 0.1) × 10^–17^ cm^2^ at 300 K, which is in an excellent agreement to the data reported in.^33^ This cross-section we used for overall excess electrons monitoring by induced absorption Δ*n* = σ_e_ × Δα(*t*) as well as to TG diffraction efficiency DE(*t*), see in Methods. On a time-scale of 10 ps laser pulse, the hole-polaron can be assumed as nonresponsive, and its contribution into induced absorption is negligible due to a very high polaron mass *m*_p_ = 18 *m*_0_.^14^

**Figure 2:**
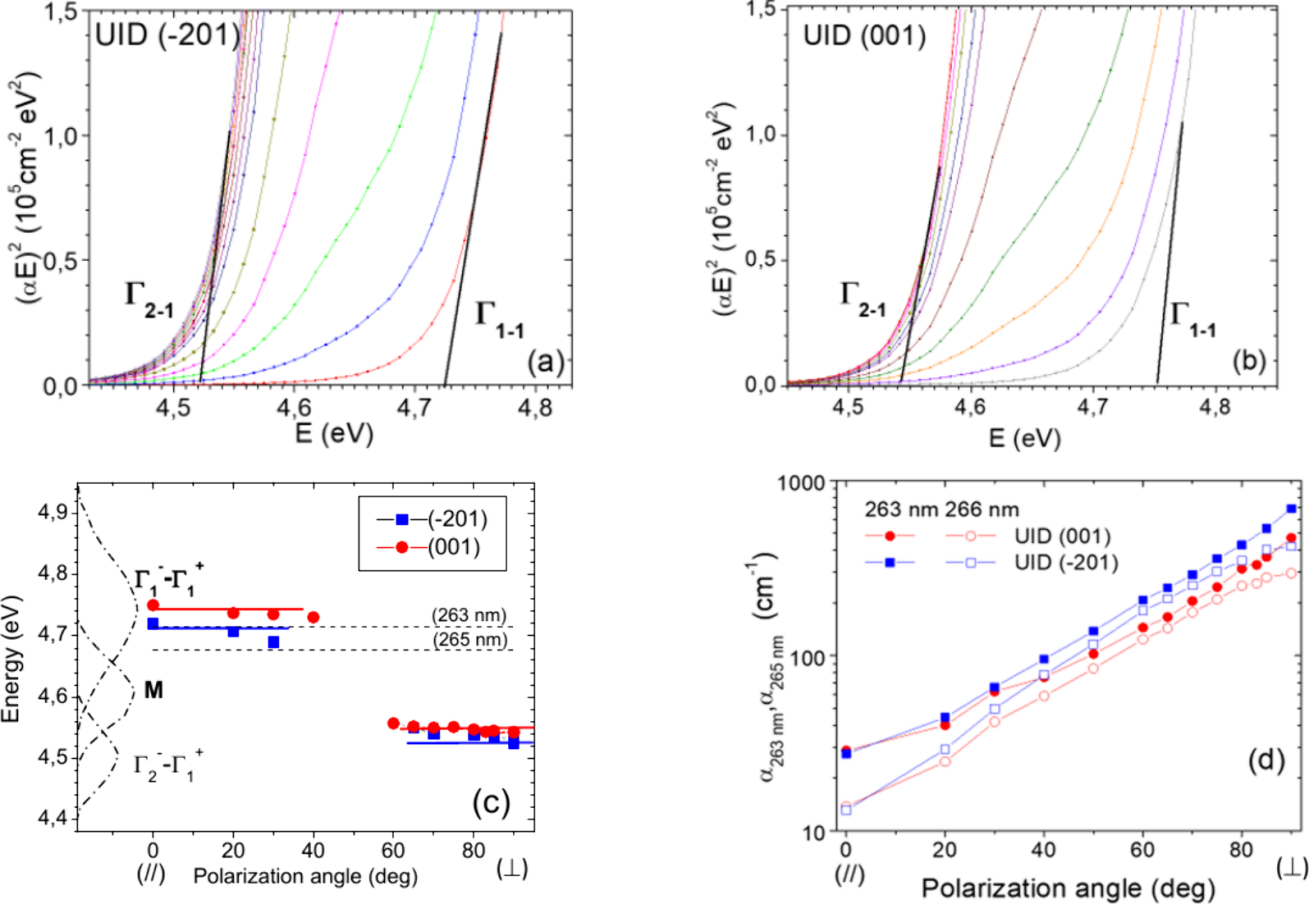
Absorption anisotropy at the bandgap in β-Ga_2_O_3_ for polarized light. (a, b) Direct band gap is extracted from the Tauc plot and given by symbols in (c). Fourth harmonic laser energies (horizontal dashed line) are used in time-resolved Δα, DE, and PL measurements. On the left side three bands (dot-dashed) aimed to eXciton excitation in (001) plane from matching data of Meißner et al.^30^ at 300 K. (d) Extracted absorption coefficient at two laser wavelengths vs the light polarization angle.

To obtain the absorption values dependent on polarization at bandgap, the samples were polished, and the α coefficient was extracted using multiple reflection formula (see the transmittance spectra in Supporting Information, Figure S2). The corresponding Tauc plots of absorption are shown in Figure 2a,b. A straight-line intersection with the *x*-axis was used to determine the direct bandgap values at 0° (//) and (⊥) polarizations as revealed in Figure 2c. These bandgap values are in good agreement with those previously reported.^8,11,30^ The two horizontal lines in Figure 2c indicate the laser energies of 263 (4.714 eV) and 266 nm (4.678 eV) for the fourth harmonic light used in the time-resolved measurements. On the left side in Figure 2c we indicate eXciton photoluminescence exitation (PLE) spectra from steady-state measurements shifted to 300 K by the peak position temperature dependence in Figure 4 of ref.^30^ The Γ_2–1_ and Γ_1–1_ band amplitudes demonstrate the opposite rotation by polarized light at 90° in a sinusoidal manner. This feature is anticipated by density functional theory (DFT) and was confirmed experimentally.^30,34^ The intermediate band M-amplitude has no polarization dependence. This band was resolved, for the first time, by Meißner et al.,^30^ and its origin theoretically is still not appointed. As can be seen from Figure 2c, at 0° (//), our laser energies can only occur involving excitation of eXcitons by the Γ_1–1_ band and the M band. At 90° (⊥), the eXciton is excited by the M-band and the Γ_2–1_ band.^30,36^ In the following, we assume that excitation is simply proportional by the corresponding absorption values at α_263 nm_ or α_266 nm_, which were measured and presented in Figure 2d as a function of the polarization angle.

### Radiative Emission

**Figure 3:**
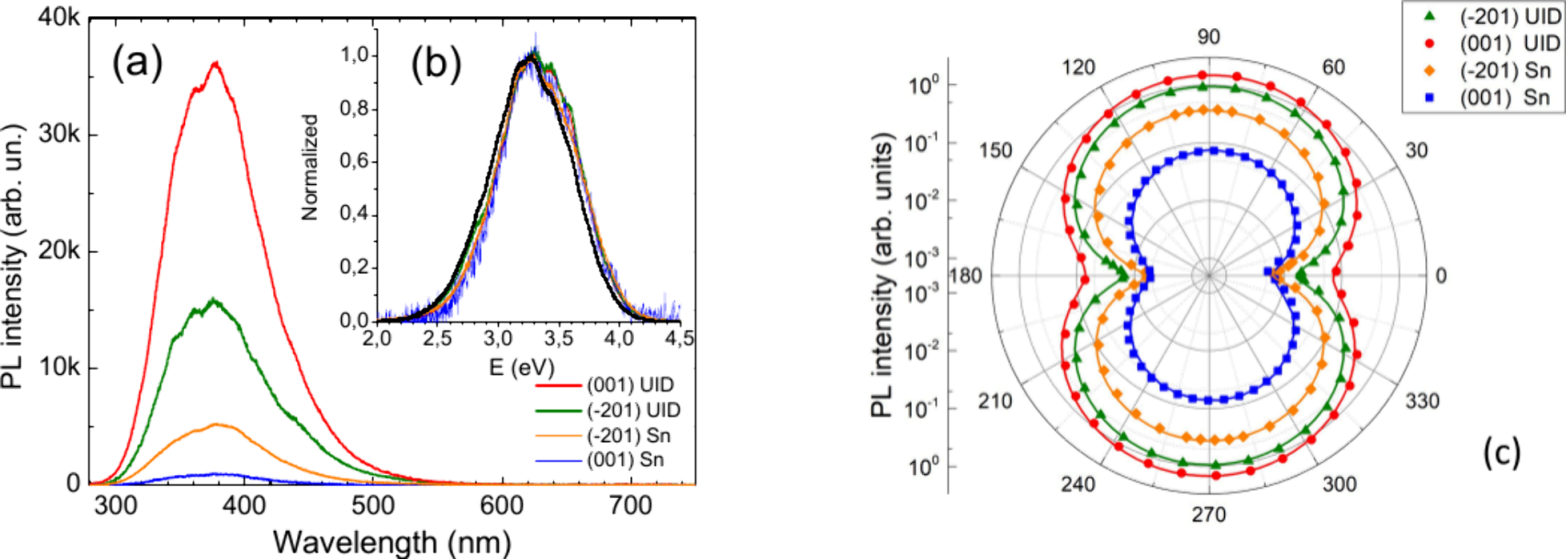
(a) Integrated PL spectra in β-Ga_2_O_3_ samples for a constant excitation at (⊥) polarization under front emission geometry and (b) the same spectra normalized to a peak magnitude at 3.35 eV in a function of quantum energy. The bandwidth is 0.77 ± 0.04 eV. (c) Polar plots of integrated eXciton emitted light in transmission geometry. Note the log-scale of the PL intensity used for the purpose of better resolution. Solid lines are sinusoidal fits.

Using 200 fs laser pulses, we conducted spectral measurements of emitted light in the front emission geometry (Figures 3a,b) and under transmission geometry (Figure 3c). In the latter case, a 2 m long optical fiber bundle was used for light collection (see Methods). The strong absorption at (⊥) polarization is typically used to prevent excitation light from passing through the sample into the detector. The recorded photoluminescence (PL) spectra (Figure 3a) were obtained at constant fluence, and spectra were corrected for the response function of the registration system. It should be noted that the eXciton band is independent of the excitation level (see time-resolved PL results in the next section). The PL peaks occur at 3.35 eV with a bandwidth of 0.69 ± 0.04 eV. Very similar results are obtained by unpolarized light for CL measurements as presented in Supporting Information Figure S3; the peak at 3.28 eV, width 0.77 ± 0.06 eV. In both cases, the measured parameters fall into the range of the reported values at 300 K. The PL spectral shape can be interpreted by two close Gauss peaks overlap with an energy difference of 0.1–0.14 eV as was done in refs.^35,38^ Such an approach is based on the assumption that hole-polaron localization could be located either on a single O(I) atom or on a shared bond of O(I)–O(II) between two oxygen atoms. The relative weight of two Gauss peaks typically is similar.^17,36,38^ Therefore, we do not provide deconvolution and, instead, adopt an equivalent property within the integrated PL band. Additionally, small ripples are observed at the top of the PL band, which are also detected in the unpolarized CL spectra Figure S3a,b. These ripples have been reported in previous works,^35^ but their origin has not been uniquely interpreted.

The evident feature from Figure 3a is that the integrated PL intensity notably decreases in samples with higher *n*_0_ concentration. This behavior implies a stronger nonradiative recombination rate as discussed later. At the same time, one can clearly note the same band profile in all samples, as witnessed by normalized plots of the quantum energy in Figure 3b. The intrinsic band quenching with *n*-type doping in β-Ga_2_O_3_ was noticed in previous reports by unpolarized PL detection.^36^ In a recent work of Cooke et al.^37^ PL polarization was also investigated under different excitation light conditions. In this context, we also have examined the PL by placing a linear analyzer, and the results are presented in Figure 3c. The figure shows that the eXciton band is very notably highly polarized; note the log-scale here for the better resolution at low intensity. The strongest emission intensity occurs under E//*a* directions in the (001) plane and under the E//[102] direction in the (−201) plane, angles 90°–270°. Weak intensity occurs under emission E//*b*, corresponding to angles 0°–180°. These findings agree with the data reported in ref.^37^ To better quantify anisotropy, we have applied a sinusoidal function for fitting in Figure 3c with the following parameter: *P* = (*I*_max_ – *I*_min_)/(*I*_max_ + *I*_min_) as shown by solid lines. The result indicates that, in the (−201) plane, the anisotropy exceeds *P* = 0.97 for the UID and Sn-doped sample. In the (001) plane, the anisotropy is *P* = 0.91 in UID and *P* = 0.86 in the Sn-doped sample, meaning slightly reduced dipole orientation. In any way, a high factor *P* guarantees that the intensity of unpolarized detection light typically follows the most emitted intensity in the line 90°–270°. This explains the unpolarized CL spectra in Figure S3a,b generally match the polarized PL spectra in Figure 3a,b.

Another feature of Figure 3a is that, under above-bandgap excitation, we do not observe supporting bands in the 400–550 nm wavelength range, which are known to emerge when a hole-polaron is localized to recharged Ga or O vacancies.^22,24^ Additionally, no DAP bands in the 650–800 nm range are observed which would signify Fe or Cr impurity contamination.^11^ In order to provide more extended mapping for the DAP we used a test at a strong confocal CW-excitation by 355 nm light (within eXciton band) by focusing into a 2 μm diameter spot projected directly on the sample surface. These results are presented in Supporting Information, Figure S4a. In these spectra, blue-green defect bands became visible at rear spots, producing bands around 412 nm (3 eV), 438 nm (2.84 eV), and approximately 500 nm (2.4–2.6 eV), consistent with DAP results observed by Wang et al.^38^ It is worth mentioning that the intensities of these PL bands do not correlate with the equilibrium electron *n*_0_ density in the samples. Furthermore, PL mapping reveals that intensities vary significantly from spot to spot (Figure S4b). In addition, we observe that the emitting places exhibit intensive degradation by prolonged CW excitation in the range of minutes. Similar degradations in β-Ga_2_O_3_ were reported in ref.,^39^ where the authors attributed them to defects located at surface structural imperfections. We think that rare defects close to the surface do not provide sufficient contribution into eXciton emission during the time-resolved measurements due to strong bulk-like absorption above the bandgap.

## Time-Resolved Investigations. Results

**Figure 4:**
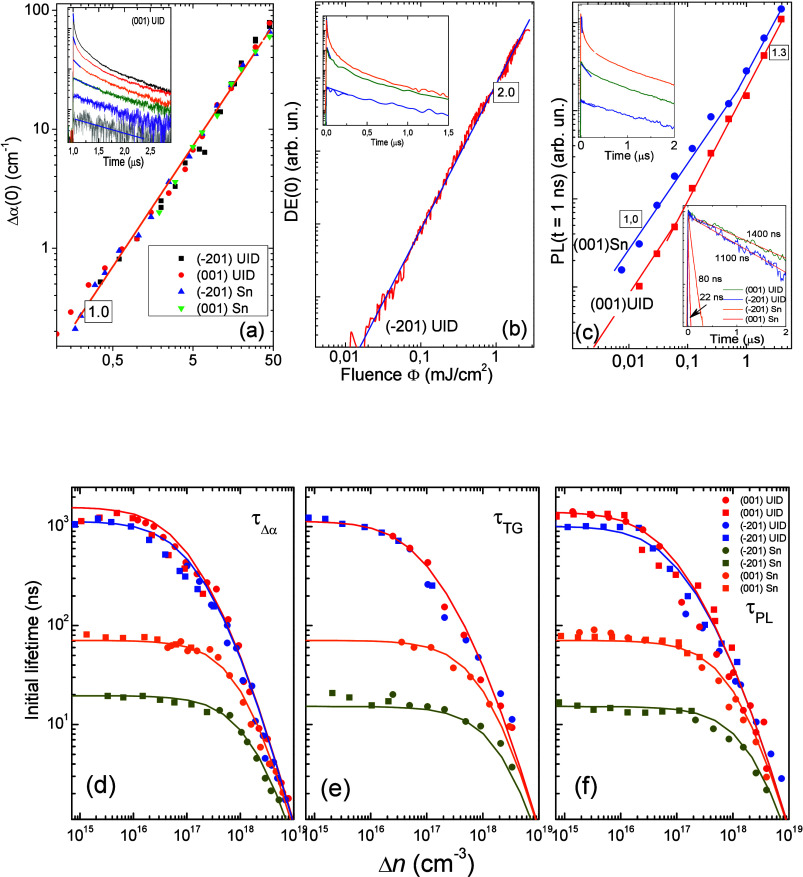
**(Upper row)** Excitation dependences of photoinduced amplitudes in β-Ga_2_O_3_ samples: (a) Δα(0), (b) DE(0) (Λ = 1.5 μm), (c) PL (*t* = 1 ns); the slope parameter is indicated. The top inset shows the corresponding transient at selected excitation. The blue lines in trace beginning indicate fit for initial decay time τ_init_ definition. The inset (c) bottom demonstrates normalized PL decays for the exponential lifetime extraction at Δ*n* ≪ *n*_0_. **(Bottom row)** τ_init_ versus average calculated density Δ*n* of the injected pulse: (d) τ_Δα_, (e) τ_DE_, and (f) τ_PL_. Symbol point color indicates the sample, and the guiding lines are given. The excitation polarization is presented either by circle (//) or by rectangular (⊥) mark.

In this section, we provide results of the induced signals and decay dynamics following different laser pulse excitation. Examples of amplitude dependence as a function of excitation level are shown in the top-row panels of Figure 4. In these measurements, (⊥) light polarization is used with distinct excitation spot size for different techniques. (This is due to variations in available power from different lasers at the fourth harmonic wavelength, resulting in slightly altered excitation geometries.) For this purpose, the scaling argument is analogous but with distinct fluence, Φ (mJ/cm^2^). Figure 4a–c illustrates the linearity of the carrier density generation versus Φ. The behavior for all samples is seen by the induced absorption amplitude Δα(0) in Figure 4a. Note that such linear dependence persists at low-level (LL) regime (where excess density is below of doping density, Δ*n* ≪ *n*_0_) as well as in high-level (HL) regime (Δ*n* ≫ *n*_0_). This linearity was also monitored under other polarizations; not shown here. Furthermore, a detailed inspection indicates that the linearity remains even under the extreme Φ values at Δ*n* ≥ 10^19^ cm^–3^. This fact justifies the absence of the Burstein-Mott dynamic shift at the absorption edge of the β-Ga_2_O_3_ bandgap. The linear generation of excess carriers ensures the observed quadratic dependence of the diffraction efficiency DE(0) presented in Figure 4b. Note that DE(0) is created by the refraction coefficient change,^40^ see eq 6 as described in eq 6 in the Methods section.

In Figure 4c we show the excitation dependence of the PL emission over eXciton band integrated between 2.7 and 4.0 eV. The PL amplitude was monitored at a 1 ns delay time after the laser pulse. The delay was employed to exclude certain fast processes with characteristic times of 90–200 ps (see Supporting Information, Figure S6). These fast processes are operating in opposite directions in samples on different planes; i.e., PL intensity slightly rises by about 20% on β-Ga_2_O_3_ (−201) plane, while it decreases of about 50–60% on β-Ga_2_O_3_ (001) plane. As shown in Figure S6, these effects are independent of the excitation level, as well as to a presence of enhanced doping. Based on these observations, we interpret them as resulting from hole-polaron delocalization between two oxygen states. Similar transients on picosecond time scale have been observed in β-Ga_2_O_3_ samples with hole-polaron localized on recharged Ga vacancies.^22,24^ In the following discussion, we will not address these fast transients, as they do not influence recombination decay on longer time scales (1 ns–10 μs). As shown in Figure 4c, PL (*t* = 1 ns) linearly increases under LL excitation; however, in the Sn-doped sample, the PL intensity is 3–4 times greater than in the UID sample. Under HL excitation, the amplitudes converge, as PL in the UID sample begins to rise more rapidly, with a slope of 1.3. A similar trend is observed in the (−201) plane of β-Ga_2_O_3_ samples (not shown here).

The upper insets in Figure 4 demonstrate the corresponding transients of those signals at selected excitations for the UID samples. By analyzing the decay profile, one may distinguish the nonexponential recombination at HL excitation, which is clearly presented in all three panels. At LL excitation, the decays exhibit a simple exponential decay. For UID samples, the exponential decays occur on μs-time scale. At LL excitation, the monitored signals show a decreasing signal-to-noise ratio, with the best sensitivity achieved for PL decay measurements. This sensitivity enables precise exponential lifetime extraction, as shown in the bottom inset of Figure 4c in the normalized plot. The extracted lifetimes exhibit a strong decrease with increasing n_0_. Numerical values are listed in Table 1.

A detailed analysis of the nonlinear recombination mechanism requires a determination of the effective carrier decay time as a function of a fixed created carrier density.^44^ In the presented case, where the laser pulse duration is being much shorter than the carrier lifetimes, the effective lifetime, τ_eff_, can be evaluated straight-forwardly from initial time constant, τ_init_. All alternative route procedures for τ_init_ extraction, in general, do not perturb model-dependent analysis on the nonlinear recombination process.^20,40−42,44−47^ In the current work, we adopted an averaging profile over numerical data points in limited time moments of the decay trace beginning. For a longer slope, we used a straight-line fit on a logarithmic scale. In both cases, we assumed that the effective carrier density reduced to the level of 1/e. The τ_init_ evolution is illustrated by examples in Figure 4a–c (upper insets, blue line). The fixed carrier density created by excitation pulse for each transient case was calculated by the incident fluence and counting the average Δ*n* value under the laser absorption depth (Supporting Information S8).

The initial times τ_init_ are presented correspondingly in the bottom row of Figure 4. Different symbol colors represent each sample, while symbol shapes indicate the two excitation polarizations. Equivalent scales are presented in Figure 4d (τ_Δα_), Figure 4e (τ_DE_), and Figure 4f (τ_PL_). However, we note potential absolute errors arising from differences in excitation spot sizes, experimental setup geometry, and excitation wavelengths. Considering these factors, we estimate an absolute error of ∼25% for the Δ*n* scale and 15–20% for the initial lifetime τ_init_. Within this error range, the results from the three measurement techniques align closely, as indicated by the applied trend lines.

The experiments represent the decay in the effective lifetime which is governed by a combination of radiative and nonradiative recombination rate,^41^
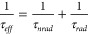
1

A nearly identical fit in Figure 4d,e and Figure 4f (radiative emission) indicates that a single term dominates in eq 1. At room temperatures, the dominant term is the nonradiative recombination rate 1/τ_nrad_.^27,30,43^ Consequently, we cannot determine the radiative rate (1/τ_rad_) directly using eq 1. The value of τ_rad_ was obtained through subsidiary measurements of internal quantum efficiency IQE in an Ulbricht sphere (see Methods, Supporting Information, S7). The IQE value η can be expresses as^42^
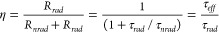
2

Despite the simplicity of this method, accurate extraction of τ_rad_ is preferable under conditions where Δ*n* ≪ *n*_0_, as τ_eff_ remains constant in this regime. In such cases, the injection dependence in the decay does not significantly affect the large τ_rad_/τ_eff_ ratio. The experimentally obtained η and τ_rad_ values are listed in Table 1. For UID samples, η = 14–17%, consistent with previously reported values of η = 5–8% at 290 K in β-Ga_2_O_3_.^43^ For Sn-doped samples, we measured reduced η values of 2.5% and 0.8% correspondingly, indicating an increased influence of nonradiative recombination.

**Figure 5:**
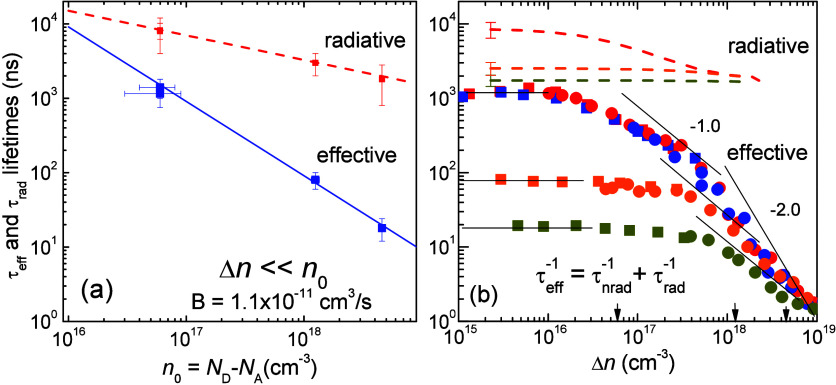
τ_nrad_ lifetimes obtained in β-Ga_2_O_3_ samples by photoinduced absorption in comparison to the extracted τ_rad_ lifetimes: (a) as a function of *n*_0_ at (Δ*n* ≪ *n*_0_), (b) as a function of Δ*n*. Used symbols are the same as those in Figure 4f. The *n*_0_ values in different samples are denoted by arrows to the bottom axis. Thin solid lines indicate expected slope for the effective lifetime.

In Figure 5 we show the τ_eff_ obtained by the single technique of the induced absorption measurements in comparison with extracted τ_rad_ lifetimes. The results for the two UID samples are very similar and can be treated nearly equivalent. In Figure 5a lifetimes are presented as a function of *n*_0_ under the condition Δ*n* ≪ *n*_0_. The effective recombination rate follows the dependence 1/τ_eff_ = B*n*_0_ with parameter B = (1.1 ± 0.15) × 10^–11^ cm^3^/s, while the radiative rate indicates a dependence which can be described empirically as 1/τ_rad_ = 0.35 × *n*_0_^1/3^ [s^–1^]. In a typical *n*-doped semiconductor the radiative recombination rate for unbound pairs in the parabolic band under Δ*n* ≪ *n*_0_ is usually described by a relation R_rad_ ∼ B_r_*n*_0_.^44,45^ Even if all carriers are bound in pairs (free-excitons), one might expect a constant radiative lifetime due to the constant charge density in exciton Bohr volume.^46,47^ In β-Ga_2_O_3_ the eXciton has a very large binding energy due to a disordered dielectric environment. If the screening of *n*_0_ can be neglected due to effective dielectric constant 3.9 obtained by calculations,^48^ the aim for radiative lifetime dependence is caused by the anisotropy of the dipole matrix elements for the eXciton.

Using the τ_rad_ obtained under LL excitation, we evaluated the dependence of τ_rad_ (Δ*n*) based on the PL peak dependence in Figure 4c. This is feasible because the PL amplitude is not significantly affected by dominant nonradiative decay. Such a procedure has been previously applied for τ_rad_ numerical estimation in GaN and in related AlGaN quantum wells (QWs).^45,50^ In this approach, we assume a linear proportionality of the generated electron Δ*n* density as observed from Figure 4a. The obtained dependences of τ_rad_ (Δ*n*) are indicated by dashed lines in Figure 5b up to Δ*n* ∼ 2 × 10^18^ cm^–3^. Beyond this excitation, the nonradiative decay influence becomes important. For the β-Ga_2_O_3_ UID samples, τ_rad_(Δ*n*) decreases by Δ*n* and approaches a constant value of the two Sn-doped samples. In a typical semiconductor at (Δ*n* = Δ*p*) ≫ *n*_0_, the free pair radiative lifetime is expected to follow the dependence 1/τ_rad_ ∼ B_rad_Δ*n*.^44,47^ The observation of a constant τ_rad_ at Δ*n* ∼ 10^18^ cm^–3^ indicates the unscreened Bohr radius of the eXciton.

A few general observations from Figure 5b are noteworthy. First, the obtained effective lifetime variation is independent of excitation polarization at the bandgap for both β-Ga_2_O_3_ planes. Second, the influence of the radiative rate on the effective recombination can be virtually neglected at any excess or doping concentration (*n*_0_ values are indicated by arrows on the Δ*n* axis in Figure 5b). Third, we can recognize at least three distinct regions for effective lifetime τ_eff_(Δ*n*) dependences, as indicated tentatively by thin-solid lines in the figure. Those regions are (i) the exponential lifetime τ_eff_ under Δ*n* ≪ *n*_0_ regime, strongly dependent on *n*_0_, (ii) a first-order dependence τ_eff_ ∼ Δ*n*^–1^ under intermediate Δ*n* ∼ *n*_0_ condition, decreasing with increasing *n*_0_, and (iii) a universal inverse quadratic dependence τ_eff_ ∼ Δ*n*^–2^ under HL regime, Δ*n* ≫ *n*_0_. This dependence is characteristic of Auger recombination processes in highly excited semiconductor materials.^47,49^ The discussion of different term interactions of these processes in β-Ga_2_O_3_ will be provided in the following Section “Modeling of Nonradiative Recombination”.

**Figure 6:**
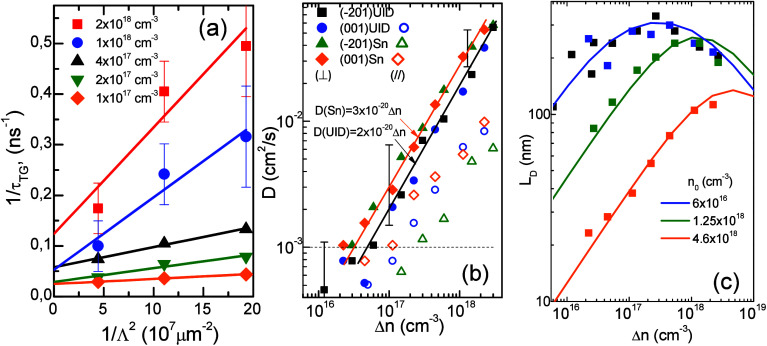
Diffusion coefficient in β-Ga_2_O_3_ as a function of Δ*n*. (a) Plot of 1/τ_TG_ vs 1/Λ^2^ in the (−201) UID sample. (b) Dependences in (⊥) direction are shown by solid symbols, and in (//) direction by open symbols. The absolute error and the limit of resolution (dotted horizontal-line) is indicated. The solid lines indicate fits for UID and Sn-doped samples in the direction (⊥). (c) The diffusion length *L*_D_ = (*D*τ_eff_(Δ*n*))^0.5^ at direction (⊥). Fitted lines were obtained by using τ_eff_(Δ*n*) obtained by a modified Auger recombination equation.

Figure 6 presents the decay results obtained from transient grating (TG) measurements at variable interference spatial periods Λ. As explained in the Methods section, the TG transient can be plotted in the coordinates 1/τ_TG_ versus 4π^2^*D*/Λ^2^ allowing the separation of the recombination lifetime (by intersect of 1/τ_TG_ at 1/Λ = 0) from the diffusion-related term, determined by the slope. The obtained results in Figure 6a present a set of the measured dependences in the (−201) UID sample in (⊥) at the crystallographic direction [102]. The applied straight line indicates an increase in the diffusion *D* coefficient that correlates with a decrease in the recombination lifetime (i.e., higher 1/τ_R_). The appropriate coefficients have been extracted by both plane measurements in β-Ga_2_O_3_ samples in two orthogonal directions. The results are shown in Figure 6b. The symbol shape presents the direction (⊥) while the open mark corresponds to the (//) direction. (Data in the (−201) UID sample at (//) not shown because they are close to the resolution limit as presented here by horizontal dashed line.) Also, the error bars are included for a reliable range of *D* definition within acceptable 1/τ_R_. As can be seen, the data suggest an anisotropy in diffusivity, which is enhanced in the (⊥) directions. Here, the diffusion coefficient is empirically approximated by thick solid lines, for the UID samples: *D*_UID_(Δ*n*) [cm^2^/s] = 2 × 10^20^Δ*n* [cm^–3^] and slightly higher for Sn-doped samples: *D*_Sn_(Δ*n*) [cm^2^/s] = 3 × 10^20^Δ*n* [cm^–3^]. In any case, diffusivity is too low to be explained by free electron diffusion. For the reported electron Hall mobility in β-Ga_2_O_3_ at 300 K of 20–100 cm^2^/(V s),^3,35,51^ the electron diffusion coefficient *D*_e_ calculated using the Einstein relation would be 0.5–2.5 cm^2^/s, which is 2 orders of magnitude higher than the measured diffusivity in the (⊥) directions and at least three orders higher in the (//) directions. Consequently, we attribute *D* to the eXciton localization properties. The diffusion process likely occurs via hole-hopping between nearby oxygen atoms, restricted by the high hole-polaron mass 18 m_0_,^14^ which limits the hopping ability. The diffusion length in the directions (⊥) was calculated as *L*_D_ = (*D* × τ_eff_)^1/2^, and it is shown by symbols in Figure 6c. The obtained *L*_D_ is negligibly small in the LL regime but grows up to 100–300 nm at intermediate excitation levels.

## Modeling of Nonradiative Recombination

To analyze the nonradiative lifetime trends, we may assume that β-Ga_2_O_3_ excited crystal consists of carrier pairs bound as eXcitons along with a residual density of free electrons. In other words, we neglect the processes of temporal self-trapped hole ionization and eXciton dissociation. Those processes are considered to be important for β-Ga_2_O_3_ Schottky diodes under high external electric fields.^14^ In the absence of an external field, both actions are short in a time range in which the effective nonradiative lifetime is determined. Then, obtained injection dependence of τ_eff_ in the current work can be reasonably reproduced using the conventional ABC recombination model of *n*-type semiconductors:^47,50^
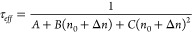
3

Here, A represents the first-order rate coefficient, commonly associated with Shockley-Read-Hall (SRH) recombination involving existing impurities, and B is the second-order rate coefficient, which, in this case, could describe trap-assisted Auger (TAA) recombination. Finally, C is the third-order rate coefficient for band-to-band *eeh* Auger recombination.^47,49,52^

Our lifetime data for UID samples suggest that recombination through the SRH mechanism is limited due to the extremely low hole (polaron) mobility ≪1 cm^2^/(V s) in β-Ga_2_O_3_.^14^ Therefore, we introduced only a small value of A = 10^5^ s^–1^ corresponding to an upper limit τ_SRH_ ∼ 10 μs. The effective lifetime is determined by bipolar parameter B = (1.1 ± 0.15) × 10^–11^ cm^3^/s as shown in Figure 5a. The nonradiative TAA process in semiconductors has a long history since a first paper published by Landsberg in 1964.^53^ During TAA recombination, the kinetic energy released when a carrier is captured by a strongly localized, high-density trap is transferred to another free carrier, exciting it into an available state in the band. TAA formation in the ultrawide bandgap material was studied theoretically in^54^ invoking the experimental results of substitutional Ca_Ga_ deep impurity in InGaN light-emission quantum wells (QWs).^50,55^ In the case of β-Ga_2_O_3_ crystal, the hole-polaron itself serves as a localized trap.^14^ Under its projected radius of ∼0.1 nm,^14,56^ the density of the positive charge in the polaron is of the order of 10^23^ cm^–3^. We attribute this to TAA *ee* processes in β-Ga_2_O_3_, where the letters represent the captured electron and the hot electron receiving the released energy.

**Figure 7:**
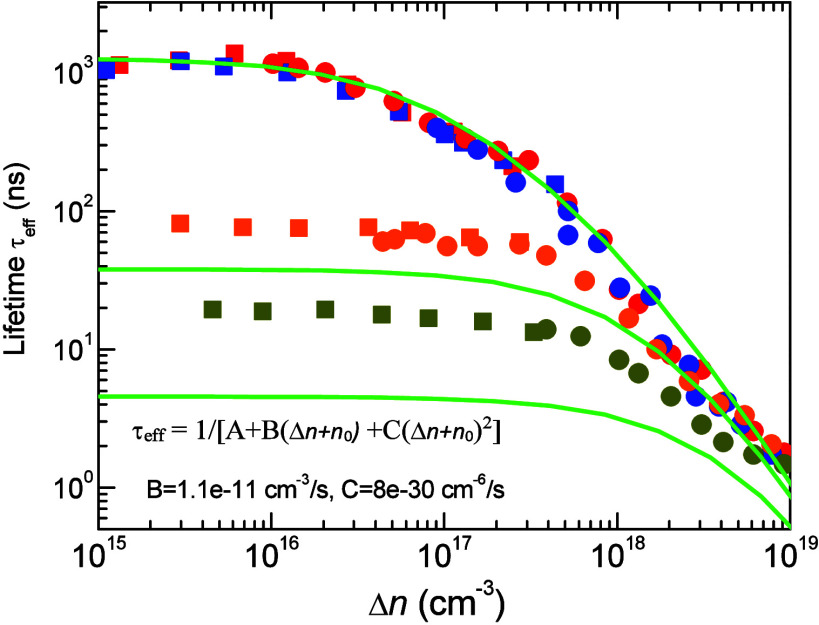
Calculated lifetimes of τ_eff_ by conventional ABC model, eq 3 with parameter B (Figure 5a) and parameter C (Figure 5b) in the full range of excitation and doping. The fitting does not allow us to obtain quantitative agreement with τ_eff_ values at LL excitation. If C parameter is enhanced or reduced, disagreement in orders occurs over other density range, regardless of C parameter choice.

The Auger parameter C = 8 × 10^–30^ cm^6^/s is derived from Figure 5b. Inserting both B and C and *n*_0_ from Table 1 into eq 3 we calculate a set of τ_eff_(Δ*n*) intermediate dependences. The simulated lines compared to the experiment are shown in Figure 7. While the fits reproduce the qualitative trends in lifetime dependence, the ABC model cannot quantitatively account for τ_eff_ values under low-level (LL) excitation. This discrepancy arises because the Auger recombination coefficient C in eq 3 depends quadratically on equilibrium density 1/C(*n*_0_)^2^. However, this relationship is not observed experimentally, as shown in Figure 5a. Adjusting C to resolve this discrepancy would lead to disagreement between the long exponential lifetimes of UID samples and the lifetimes observed in the high-level (HL) regime.

Among possible assumptions for a better fit, we should consider the variation in the Auger recombination coefficient due to the electron–hole density correlation within the eXciton Bohr volume. In general terms, these correlations should include some degree of Pauli anticorrelation caused by electron–electron repulsion. A common impact of these effects was described on various levels of many-particle theory by introducing the “enhancement factor” formalism proposed in.^47,52^ Then the measured values of the Auger coefficient can be presented as C = g_eeh_C_0_ where C_0_ is the Auger coefficient without *e-h* correlations and g_eeh_ is an effective factor due to common attraction/repulsion averaging. In the case of Wannier-Mott free-exciton, which is formed from two independent particles by chemical reaction (according to the balance described by the Saha equation^57^) the theory predicts that enhancement factors increase under low screening conditions. For the exciton binding energies 20–50 meV, the calculation yields maximal g_eeh_ values 50 to 1000.^41^ These values are comparable to experimental results observed in bulk (3D) semiconductors (like Ge, Si, 4H-SiC), in the (2D) AlGaN QWs systems^47^ and in (0D) CdSe nanocrystals.^58^ For the eXciton in β-Ga_2_O_3_ which is weakly screened, g_eeh_ could be assumed constant. If we choose C_0_ ∼ 10^–31^ cm^6^/s as a typical value of phonon-assisted Auger transitions in a wide-band gap semiconductor,^59^ then, based on the measured C = 8 × 10^–30^ cm^6^/s, the estimated g_eeh_ value would be approximately 80. If eXcitons experience an increase in C at low Δ*n* densities (due to increasing g_eeh_^41^), this would result in a significant discrepancy between the estimated and observed τ_eff_(Δ*n*) dependencies, regardless of the choice of C_0_. Furthermore, eXciton screening is not evident in the radiative lifetime presented in Figure 5b. For a typical Wannier-Mott exciton, this lifetime should similarly reflect an enhancement B_rad_ = g_eh_B_r_.^60^

To refine the ABC model further and achieve better agreement with experimental data, we propose adopting a more unified approach to the Auger recombination process in β-Ga_2_O_3_. In a generalized sense, the isolated *eeh* Auger process from eq 3 can be rewritten without including other terms as follows:
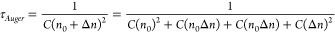
4

In the right-hand side of eq 4 the first term in the denominator is proportional to ∼ C(*n*_0_)^2^. However, the experiment in Figure 5a displays ∼ B(*n*_0_) for the TAA process. The last term in eq 4 has a proportionality to ∼ (Δ*n*)^2^ and is most conspicuous in high excitations range (Figure 5b). Note that two intermediate terms contain mixed proportionality to *n*_0_Δ*n* and obviously have a pronounced effect at intermediate excitations *n*_0_ ∼ Δ*n*. It is important that both terms contain the dependence ∼ (Δ*n*)^1^ in weighting to *n*_0_. In these constraints, it is worthwhile to consider that one part of the intermediate recombination rate could be represented by TAA recombination as BΔ*n* and the other part by the third-order Auger process as C*n*_0_Δ*n*, as indicated by thin lines in Figure 5b of the Δ*n*^–1^ proportionality. Thus, including the A parameter, the effective lifetime can be rewritten in the following form:
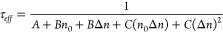
5

**Figure 8:**
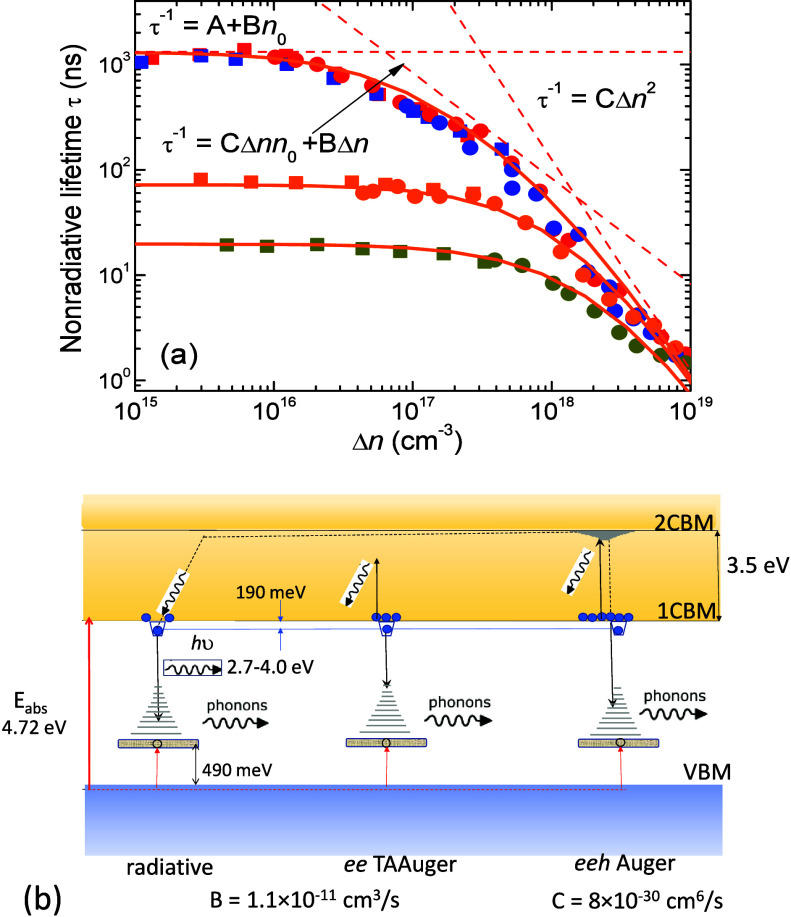
(a) Lifetimes, τ_eff_, calculated using the ABC modified model described by eq 5 with the same parameters as those used in Figure 7. The dashed lines guide the contribution of three Δ*n*-dependences to the fit for UID samples, *n*_0_ = 6 × 10^16^ cm^–3^. (b) Diagram explaining radiative and two kinds of Auger processes in the presence of the eXciton. The TAA process described by B parameter takes place due to a localized hole-polaron. The going on *eeh* Auger process is described by the C parameter. Different final states of hot electron and amount of released energy taken by phonons could be expected.

By eq 5 we propose the ABC modified model, which does not require any other terms to be included. The equation assumes only the two kinds of Auger processes, which replace each other as the excitation level increases. The data calculated by eq 5 are shown by solid lines in Figure 8 with the same parameters used as in Figure 7. The excellent agreement to the experimental data is now obtained. As shown for the UID sample, there are three Δ*n*-dependences drawn by dashed lines and guiding the contributions. The transition between the two Auger processes occurs within the interval Δ*n* = 10^17^–10^18^ cm^–3^. As can be seen in Figure 6b, a steep increase in eXciton diffusion takes place in this excitation interval. This correlation implies that the Auger recombination mechanism could be associated with the ability of the exciton to diffuse, a process stimulated by hole hopping. At low excitation, the eXciton behaves as a deep localized trap, a condition warranted by TAA processes. At high excitation, this condition is lost and Auger recombination increases due to movement of the eXciton into a three-particle *eeh* Auger mechanism. The enhancement factors are included in the B and C parameters.

The two Auger and one radiative process for the β-Ga_2_O_3_ band gap are illustrated schematically in Figure 8b. The eXciton is created nearly instantly through hole-self-trapping to a polaron state with an average energy of ∼490 meV, while the binding energy of the electron to eXciton is ∼190 meV.^14^ Radiative recombination occurs under a configuration diagram shift influenced by the strong ELC effect. The electron transition to the ground state includes vibrational broadening, producing a wide PL band in the 2.7–4.0 eV range. The PL radiation is highly anisotropic due to the orientation of the allowed dipoles. The Auger recombination determines the effective electron lifetime. In LL and intermediate excitation, the Auger process occurs mainly as a TAA transition where hole-polaron is acting as a deep, unmovable trap. At HL excitation, this process transforms into three particle *eeh* Auger transitions where the ability of the eXciton density correlation is included in the obtained parameter C. The ABC modified model, however, does not involve diffusion anisotropy of the eXciton. Therefore, a change in Auger mechanism very likely involves the energy amount taken by a hot carrier and different initial and final energy states. The hot carriers can reach excited eXciton states below the second conduction band minimum (2CBM). Similar to radiative emission, the Auger transitions also produce strong release of ELC. If the presented hypothesis of two kinds of Auger transitions is correct, we could state that the β-Ga_2_O_3_ structure is highly unique. This is because the same intrinsic quasiparticle (eXciton) simultaneously drives three distinct processes: radiative recombination and two mechanisms of Auger processes. To the best of our knowledge, such an unusual feature has not been observed before in isotropic semiconductors.

## Conclusions

The carrier relaxation properties of wide bandgap anisotropic β-Ga_2_O_3_ crystals remain poorly explored and understood. In this work, we investigated these properties by combining three time-resolved techniques, providing detailed insight into the specific optoelectronic properties of β-Ga_2_O_3_. At room temperature, the nonradiative processes involving eXcitons are considerably faster than intrinsic radiative processes. This inequality holds over a wide range of excitations and doping concentrations. We demonstrate that eXcitons play a critical role in Auger nonradiative transitions. Simultaneously, eXciton diffusion increases and exhibits significant crystalline anisotropy at intermediate excitation levels. The conventional ABC model which includes Auger recombination fails to describe observed nonradiative lifetime dependences of free carrier concentration. By modifying the model by two Auger mechanisms: the TAA as *ee* second-order process at intermediate injections, and *eeh* third-order Auger process at high level injections, we obtain an excellent agreement with the experimental lifetime results. We also observe that the Auger recombination mechanism may be facilitated by hole-polaron hopping. These transformations, taking place by eXciton in β-Ga_2_O_3_ matrix, however, were never observed in other semiconductors before. Further research is needed to explore this unique feature of self-trapped exciton emission in metal-oxide semiconductors and its potential implications for optoelectronic devices.

## Methods

### Absorption

The VIS-IR optical transmission spectra were measured using a PerkinElmer Lambda 950 UV-NIR absorption spectrometer equipped with polarization in normal direction to the surface optical elements. The absorption coefficient α is defined from an overall transmission, taking into account sample thickness and multiple internal reflections. We used a value of constant reflectivity R = 0.16 in the extended spectral range. The reflectivity variation, however, was detected for (//) and (⊥) excitation’s polarization in energies around the bandgap and polarization angle at about 10 deg. Despite the fact that small (between 0.12 to 0.18) variations in R can generate a need for α correction in low absorption the error for stronger absorption (α ≥ 20 cm^–1^) does not exceed 5% and was not taken into account for absorption values presented in Figure 2d.

### CL Emission

The measurements were performed in the CW-mode in the spectral range of 200–900 nm with spectral resolution of 0.2 nm by using a homemade spectrometer. CL was excited with an electron beam of energy 10 keV with current of 0.5 μA focused on the sample surface to a spot of 1 mm in diameter. The spectra were taken in a single channel regime; a typical time for recording one spectrum was about 15 min. Measurements of different samples were performed at nearly identical parameters of excitation. The spectrum was corrected by taking into account the response function of the photomultiplier.

The *integrated PL* spectra were determined after excitation by 200 fs laser pulses at 265 or 266 nm with repetition rate of 10 kHz. The light collection was performed at a perpendicular direction to the sample’s backside surface using a *Hamamatsu* photonic multichannel analyzer (PMA-12) and the BBO Glan-prism analyzer placed at ∼5 cm distance from the sample. A 1 mm pinhole was positioned before the Glan prism to minimize the collection angle to protect results against possible scattered excitation. Another pinhole of 0.8 mm diameter was mounted on the sample backside to prevent transmission of the scattering light from the sample edges into the detection system. The high absorption at (⊥) polarization is used to reduce the impact from the excitation light entering through the sample into the fiber and spectrometer. Analyzer prism rotation allowed determination of optical anisotropy of the emitted light intensity.

CW-*PL* and it mapping on the sample surface were recorded by *Nanofinder-HE* apparatus in a confocal measurement geometry under reflection.^61^ Excitation light produced by the 355 nm diode laser was focused to the 2 μm diameter spot on a sample surface. The unpolarized PL light was collected. The same equipment was used for the micro-Raman scattering measurements, using 532 nm for excitation.

### Time-Resolved Experiments

*The pump–probe (PP)* measurements providing decays of induced absorption by excitation were done with 10 ps optical pulses at λ_pump_ = 263 nm from a frequency quadrupled Q-switched Nd:YLF laser (PL2243, *Ekspla*) at a 10 Hz repetition rate. The excitation’s polarization was rotated by a half wave plate. A single-mode CW laser of λ_probe_ = 1550 nm light was used at <50 mW power (*Eblana Photonics*). The probe was recorded from the center of the excited spot, which was typically of 100 μm in diameter in 18 deg angle to the surface normal. The time-resolution was equal to 100 ps using a 5 GHz bandwidth InGaAs photodetector (*Thorlabs* DET08CFC/M). Weak DT signals were amplified by a 4 GHz bandwidth low noise homemade amplifier. All transmission traces were recorded by a 6 GHz LeCroy *SDA 6000*. The decay kinetics were averaged for 50–100 traces with and without the excitation and then transferred to computer for Δα calculation.^62^ The absorption cross section of the electron was determined by a dimensionless relation *DT* = σ_e_F_0_/*h*ν where σ_e_, F_0_, and *h*ν are the electron absorption cross section, excitation fluence, and pump quant energy, respectively. The value of σ_e_ = 1.0 × 10^–17^ cm^2^ is used for doped and excited samples.

*Time-resolved PL* measurements were performed in a backscattering geometry using a *Hamamatsu* streak camera (C10627) attached to *Acton* monochromator. For excitation, 200 fs pulses with a repetition rate of 4–10 kHz from an *ORPHEUS* parametric amplifier, pumped by a *PHAROS* laser, were used. The response function is limited to 80 ps due to laser pulse jitter. The laser second harmonic at 530 nm was applied for generating the second harmonic at 265 nm. Excitation intensity was attenuated continuously by two variable neutral density filters (round continuous type, OD: 0.04–2.0).

The *internal quantum efficiency* (IQE) η of the eXciton band was measured using an integrating sphere (*AvaSphere-150*) with a waveguide spectrometer *ASEQ Instruments* LR1-T (200–1100 nm) at excitation wavelength of 265 nm, using the fs-laser running at a 10 kHz repetition rate. The excitation spot was defocused to diameter of 4 mm to reduce intensity for transfer into the LL regime. Thus, η is obtained at Δ*n* ≪ *n*_0_ where the effective lifetime is constant (Figure 5b); spectra are shown in Supporting Information Figure S7. The obtained η is used for τ_rad_ (Δ*n*) calibration, which at higher optical excitation is obtained using dependence of PL amplitude and linear carrier injection with the laser fluence.

The recombination and diffusivity parameters were measured by a light-induced *transient grating* (TG) method utilizing 10 ps Nd:YLF laser (PL2243, *Ekspla*) at a fourth harmonic of 263 nm wavelength. Two split beams produced an interference fringe pattern with well-defined spacing period Λ. The 1053 nm probe pulse of the first harmonic light was diffracted on such an excited interference pattern. The probe pulse diffraction efficiency (DE) is monitored with the help of an optical 12 ns delay-line (*Aerotech* ACT115DL). For measurement with longer delays, above 10 ns, the picosecond pump laser was synchronized with another probe pulse of 2 ns duration laser operating at 1064 nm wavelength (*Ekspla* NL202). Then, the delay is controlled electronically by a digital generator (*Highland Technology* P400) with 2 ns time resolution. The diffusion anisotropy was measured by rotating the sample to 90° in respect to the interference fringe alignment with simultaneous rotating polarization of the pump. The efficiency, typically of the order <5%, is described by the relative ratio of the probe beam transmission: DE = *I*_diff_/*I*_T_ ∼ exp(−2*t*/τ_G_), where τ_G_ is the grating decay time which contains terms of recombination and diffusion (eq 6). The τ_D_ follows from the general relation of 1/τ_D_ = 4π^2^*D*/Λ^2^. Then, by applying different Λ values with constant excitation, the diffusion coefficient *D* can be defined by the slope of the straight line. It should be noted that in β-Ga_2_O_3_ the diffraction efficiency is determined by refraction index defined by excess electrons with fitted electron mass *m*_e_* = 0.18 ± 0.05, which is much smaller of the hole-poleron mass *m*_h_* = 18.^14^
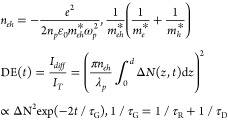
6

Here *n*_eh_ is the induced refractive index; *n*_p_, ω_p_, λ_p_ are refractive index, frequency, and wavelength of the probe light, m_eh_* is relative effective mass, ε_0_ is dielectric constant, Δ*N*(z,*t*) is excited free carrier density, and τ_G_, τ_R_, and τ_D_ are corresponding lifetimes related to transient grating, to carrier recombination, and to carrier diffusion between interference fringes.

## Supporting Information Available

Raman scattering example, optical transmission spectra at the absorption edge under different polarization of light, unpolarized CL spectra, PL mapping by confocal CW excitation at 355 nm light and examples of it is degradation, time-resolved fast PL decays on samples with different plain orientation, IQE measurement spectra by integrated PL in a sphere, average concentration definition (PDF)

## References

[ref1] Tadjer M. J. Toward gallium oxide power electronics. Science 2022, 378, 724–725. 10.1126/science.add2713. 36395206

[ref2] McCluskey N. D. Point defects in Ga_2_O_3_. J. Appl. Phys. 2020, 127, 101101 10.1063/1.5142195.

[ref3] Seryogin G.; Alema F.; Valente N.; Fu H.; Steinbrunner E.; Neal A. T.; Mou S.; Fine A.; Osinsky A. MOCVD growth of high purity Ga_2_O_3_ epitaxial films using trimethylgallium precursor. Appl. Phys. Lett. 2020, 117, 262101 10.1063/5.0031484.

[ref4] Islam M. M.; Liedke M. O.; Winarski D.; Butterling M.; Wagner A.; Hosemann P.; Wang Y.; Uberuaga B.; Selim F. A. Chemical manipulation of hydrogen induced high p-type and n-type conductivity in Ga_2_O_3_. Sci. Rep. 2020, 10, 6134 10.1038/s41598-020-62948-2. 32273592 PMC7145873

[ref5] Su J.; Guo R.; Lin Z.; Zhang S.; Zhang J.; Chang J.; Hao Y. Unusual electron and optical properties of two-dimensional Ga_2_O_3_ predicted by density functional theory. J. Chem. Chem. C 2018, 122, 24592–24599. 10.1021/acs.jpcc.8b08650.

[ref6] Min J. H.; Li K. H.; Kim Y. H.; Min J. W.; Kang C. H.; Kim K. H.; Lee J. S.; Lee K. J.; Jeong S. M.; Lee D. S.; Bae S. Y.; Ng T. K.; Ooi B. S. Toward large-scale Ga_2_O_3_ membranes via quasi-Van der Waals epitaxy on epitaxial graphene layers. ACS Appl. Mater. & Interfaces 2021, 13, 13410–13418. 10.1021/acsami.1c01042. 33709688 PMC8041250

[ref7] Ueda N.; Hosono H.; Waseda R.; Kawazoe H. Anisotropy of electrical and optical properties in β-Ga_2_O_3_ single crystals. Appl. Phys. Lett. 1997, 71, 933–935. 10.1063/1.119693.

[ref8] Ricci F.; Boschi F.; Baraldi A.; Filippetti A.; Higashiwaki M.; Kuramata A.; Fiorentini V.; Fornari R. Theoretical and experimental investigation of absorption anisotropy in β-Ga_2_O_3_. J. Phys.: Condens. Matter. 2016, 28, 224005 10.1088/0953-8984/28/22/224005. 26952789

[ref9] Zhang Y.; Xing F. Anisotropic optical and electric properties of β-Ga_2_O_3_. J. Semicond. 2023, 44, 071801 10.1088/1674-4926/44/7/071801.

[ref10] Onuma T.; Saito S.; Sasaki K.; Goto K.; Masui T.; Yamaguchi T.; Honda T.; Kuramata A.; Higashiwaki M. Temperature-dependent exciton resonance energies and their correlation with IR-active optical phonon modes in β-Ga_2_O_3_ single crystals. Appl. Phys. Lett. 2016, 108, 101904 10.1063/1.4943175.

[ref11] Sun R.; Ooi Y. K.; Dickens P. T.; Lynn K. G.; Scarpulla M. A. On the origin of red luminescence from iron-doped β-Ga_2_O_3_ bulk crystals. Appl. Phys. Lett. 2020, 117, 052101 10.1063/5.0012967.

[ref12] Yamaoka S.; Nakayama M. Evidence for formation of self-trapped excitons in a β-Ga_2_O_3_ single crystal. Phys. Stat. Sol. C 2016, 13, 93–96. 10.1002/pssc.201510124.

[ref13] Lukman I.; Bergman L. The nonradiative properties of self-trapped holes in ultra-wide bandgap gallium oxide film. Phys. Stat. Sol. B 2024, 261, 2300590 10.1002/pssb.202300590.

[ref14] Adnan M. M.; Verma D.; Xia Z.; Kalarickal N. K.; Rajan S.; Myers R. C. Spectral measurement of the breakdown limit of β-Ga_2_O_3_ and tunnel ionization of self-trapped excitons and holes. Phys. Rev. Appl. 2021, 16, 034011 10.1103/PhysRevApplied.16.034011.

[ref15] Verma D.; Adnan M. M. R.; Dhara S.; Sturm C.; Rajan S.; Myers R. C. Anisotropic excitonic photocurrent in β-Ga_2_O_3_. Phys. Rev. Mater. 2023, 7, L061601 10.1103/PhysRevMaterials.7.L061601.

[ref16] Varley J. B.; Janotti A.; Franchini C.; Van de Walle C. G. Role of self-trapping in luminescence and p-type conductivity of wide-band-gap oxides. Phys. Rev. B 2012, 85, 081109(R) 10.1103/PhysRevB.85.081109.

[ref17] Frodason Y. K.; Galeckas A.; Olsen V. S.; Weiser P. M.; Galazka Z.; Van De Walle C. G.; Vines L. Intrinsic origins of broad luminescence in melt-grown ZnGa_2_O_4_ single crystals. Phys. Rev. Mater. 2024, 8, 094604 10.1103/PhysRevMaterials.8.094604.

[ref18] L Cheng L.; Zhu Y.; Wang W.; Zheng W. Strong electron-phonon coupling in β-Ga_2_O_3_: a huge broadening of self-trapped exciton emission and a significant red shift of the direct bandgap. J. Phys. Chem. Lett. 2022, 13, 3053 10.1021/acs.jpclett.2c00682. 35352556

[ref19] Yamaoka S.; Furukawa Y.; Nakayama M. Initial process of photoluminescence dynamics of self-trapped excitons in a β-Ga_2_O_3_ single crystal. Phys. Rev. B 2017, 95, 094304 10.1103/PhysRevB.95.094304.

[ref20] Marcinkevičius S.; Speck J. S. Ultrafast dynamics of hole self-localization in β-Ga_2_O_3_. Appl. Phys. Lett. 2020, 116, 132101 10.1063/5.0003682.

[ref21] Kananen B. E.; Giles N. C.; Halliburton L. E.; Foundos G. K.; Chang K. B.; Stevens K. T. Self-trapped holes in β-Ga_2_O_3_ crystals. J. Appl. Phys. 2017, 122, 215703 10.1063/1.5007095.

[ref22] Koksal O.; Tanen N.; Jena D.; Xing H.; Rana F. Measurement of ultrafast dynamics of photoexcited carriers in β -Ga_2_O_3_ by two-color optical pump-probe spectroscopy. Appl. Phys. Lett. 2018, 11, 252102 10.1063/1.5058164.

[ref23] Cho J. Bin; Jung G.; Kim K.; Kim J.; Hong S. K.; Song J. H.; Jang J. I. Highly asymmetric optical properties of β-Ga_2_O_3_ as probed by linear and nonlinear optical excitation spectroscopy. J. Phys. Chem. C 2021, 125, 1432–1440. 10.1021/acs.jpcc.0c08413.

[ref24] Singh A.; Koksal O.; Tanen N.; McCandless J.; Jena D.; Xing H.; Peelaers H.; Rana F. Ultrafast dynamics of gallium vacancy charge states in β-Ga_2_O_3_. Phys. Rev. Research 2021, 3, 023154 10.1103/PhysRevResearch.3.023154.

[ref25] Hommedal Y. K.; Frodason Y. K.; Galeckas A.; Vines L.; Johansen K. M. H. Broad luminescence from Zn acceptors in Zn doped β-Ga_2_O_3_. APL Mater. 2024, 12, 021109 10.1063/5.0190156.

[ref26] Yanagida T.; Okada G.; Kato T.; Nakauchi D.; Yanagida S. Fast and high light yield scintillation in the Ga_2_O_3_ semiconductor material. Appl. Phys. Express 2016, 9, 042601 10.7567/APEX.9.042601.

[ref27] Tang H.; He N.; Zhu Z.; Gu M.; Liu B.; Xu J.; Xu M.; Chen L.; Liu J.; Ouyang X. Temperature-dependence of X-ray excited luminescence of β-Ga_2_O_3_ single crystals. Appl. Phys. Lett. 2019, 115, 071904 10.1063/1.5110535.

[ref28] Kranert C.; Sturm C.; Schmidt-Grund R.; Grundmann M. Raman tensor elements of β-Ga_2_O_3_. Sci. Rep. 2016, 6, 35964 10.1038/srep35964. 27808113 PMC5093899

[ref29] Feng G.; Li S.; Tian Y.; Qi S.; Guo D.; Tang W. 2 in. bulk β-Ga_2_O_3_ single crystals grown by EFG method with high wafer-scale quality. ACS Omega 2024, 9, 22084 10.1021/acsomega.4c00405. 38799343 PMC11112554

[ref30] Meißner M.; Bernhardt N.; Nippert F.; Janzen B. M.; Galazka Z.; Wagner M. R. Anisotropy of optical transitions in β-Ga_2_O_3_ investigated by polarized photoluminescence excitation spectroscopy. Appl. Phys. Lett. 2024, 124, 152102 10.1063/5.0189751.

[ref31] Peelaers H.; Van De Walle C. G. Phonon- and charged-impurity-assisted indirect free-carrier absorption in Ga_2_O_3_. Phys. Rev. B 2019, 100, 081202(R) 10.1103/PhysRevB.100.081202.

[ref32] Redley B. K. Quantum processes in Semiconductors, 3^rd^ ed., Oxford University Press; Inc.: NY, 1993.

[ref33] A Singh A.; Koksal O.; Tanen N.; McCandless J.; Jena D.; Xing Grace H.; Peelaers H.; Rana F. Intra- and inter-conduction band optical absorption processes in β-Ga_2_O_3_. Appl. Phys. Lett. 2020, 117, 072103 10.1063/5.0016341.

[ref34] Sturm C.; Schmidt-Grund R.; Kranert C.; Furthmüller J.; Bechstedt F.; Grundmann M. Dipole analysis of the dielectric function of color dispersive materials: Application to monoclinic Ga_2_O_3_. Phys. Rev. B 2016, 94, 035148 10.1103/PhysRevB.94.035148.

[ref35] Pearton S. J.; Yang J.; Cary P. H.; Ren F.; Kim J.; Tadjer M. J.; Mastro M. A. A review of Ga_2_O_3_ materials, processing, and devices. Appl. Phys. Reviews 2018, 5, 011301 10.1063/1.5006941.

[ref36] Cheng L.; Wu Y.; Zhong W.; Chen D.; Qi H.; Zheng W. Photophysics of β-Ga_2_O_3_: Phonon polaritons, exciton polaritons, free-carrier absorption, and band-edge absorption. J. Appl. Phys. 2022, 132, 185704 10.1063/5.0118843.

[ref37] Cooke J.; Lou M.; Scarpulla M. A.; Sensale-Rodriguez B. Polarized photoluminescence from Sn, Fe, and unintentionally doped β-Ga_2_O_3_. J. Vac. Sci. Technol. A 2024, 42, 022801 10.1116/6.0003216.

[ref38] Wang Y.; Dickens P. T.; Varley J. B.; Ni X.; Lotubai E.; Sprawls S.; Liu F.; Lordi V.; Krishnamoorthy S.; Blair S.; Lynn K. G.; Scarpulla M.; Sensale-Rodriguez B. Incident wavelength and polarization dependence of spectral shifts in β-Ga_2_O_3_ UV photoluminescence. Sci. Rep. 2018, 8, 18075 10.1038/s41598-018-36676-7. 30584263 PMC6305385

[ref39] Huso J.; McCluskey M. D.; Yu Y.; Islam M. M.; Selim F. Localized UV emitters on the surface of β-Ga_2_O_3_. Sci. Rep. 2020, 10, 21022 10.1038/s41598-020-76967-6. 33273495 PMC7712825

[ref40] Ščajev P.; Jarašiu̅nas K.; Leach J. Carrier recombination processes in Fe-doped GaN studied by optical pump-probe techniques. J. Appl. Phys. 2020, 127, 245705 10.1063/5.0009258.

[ref41] Hangleiter A.; Langer T.; Gerhard M.; Kalincev D.; Kruse A.; Bremers H.; Rossow U.; Koch M. Efficiency droop in nitride LEDs revisited: Impact of excitonic recombination processes. Gallium Nitride Materials and Devices X, Proceedings of SPIE, 2015; Vol. 9363, 93631R 10.1117/12.2078803.

[ref42] Hangleiter A.; Langer T.; Henning P.; Ketzer F. A.; Bremers H.; Rossow U. Internal quantum efficiency of nitride light emitters: A critical perspective. Gallium Nitride Materials and Devices XIII, ed. by Chyi J.-I.; Fujioka H.; Morkoç H. Proc. of SPIE 2018, Vol. 10532, 105321P 10.1117/12.2290082.

[ref43] Lukman I.; Bergman L. The nonradiative properties of self-trapped holes in ultra-wide bandgap Gallium oxide film. Phys. Stat. Sol. B 2024, 261, 2300590 10.1002/pssb.202300590.

[ref44] Grivickas V.; Linnros J. Carrier Lifetime: Free Carrier Absorption, Photoconductivity and Photoluminescence. In Characterization of Materials, 2nd ed.; Kaufmann E. N., Ed.; John Viley & Sons, 2012; Vol. 1, pp 658–692 10.1002/0471266965.com037.pub2.

[ref45] Langer T.; Jönen H.; Kruse A.; Bremers H.; Rossow U.; Hangleiter A. Strain-induced defects as nonradiative recombination centers in green-emitting GaInN/GaN quantum well structures. Appl. Phys. Lett. 2013, 103, 022108 10.1063/1.4813446.

[ref46] Langer T.; Klisch M.; Ketzer F. A.; Jönen H.; Bremers H.; Rossow U.; Meisch T.; Scholz F.; Hangleiter A. Radiative and nonradiative recombination mechanisms in nonpolar and semipolar GaInN/GaN quantum wells. Phys. Stat. Sol. B 2016, 253, 133–139. 10.1002/pssb.201552353.

[ref47] Hangleiter A. Recombination dynamics in GaInN/GaN quantum wells. Semicond. Sci. Technol. 2019, 34, 073002 10.1088/1361-6641/ab2788.

[ref48] Bechstedt F.; Furthmüller J. Influence of screening dynamics on excitons in Ga_2_O_3_ polymorphs. Appl. Phys. Lett. 2019, 114, 122101 10.1063/1.5084324.

[ref49] Grivickas V.; Linnros J.; Galeckas A.; Bikbajevas V. Relevance of the exciton effect on ambipolar transport and Auger recombination in silicon at room temperature, in Proc. ICPS-23; World Scientific, Singapore, Vol. 1 (1996) 91–94.

[ref50] Espenlaub A. C.; Myers D. J.; Young E. C.; Marcinkevičius S.; Weisbuch C.; Speck J. S. Evidence of trap-assisted Auger recombination in low radiative efficiency MBE-grown III-nitride LEDs. J. Appl. Phys. 2019, 126, 184502 10.1063/1.5096773.

[ref51] Zhang J.; Shi J.; Qi D. C.; Chen L.; Zhang K. H. L. Recent progress on the electronic structure, defect, and doping properties of Ga_2_O_3_. APL Mater. 2020, 8, 020906 10.1063/1.5142999.

[ref52] Hangleiter A. Recombination of correlated electron-hole pairs in two-dimensional semiconductors. Phys. Rev. B 1993, 48, 9146–9148. 10.1103/PhysRevB.48.9146. 10007142

[ref53] Landsberg P. T.; Rhys-Roberts C.; Lal P. Auger recombination and impact ionization involving traps in semiconductors. Proc. Phys. Soc. 1964, 84, 915 10.1088/0370-1328/84/6/311.

[ref54] Zhao F.; Turiansky M. E.; Alkauskas A.; Van De Walle C. G. Trap-Assisted Auger-Meitner Recombination from First Principles. Phys. Rev. Lett. 2023, 131, 056402 10.1103/PhysRevLett.131.056402. 37595230

[ref55] Myers D. J.; Espenlaub A. C.; Gelzinyte K.; Young E. C.; Martinelli L.; Peretti J.; Weisbuch C.; Speck J. S. Evidence for trap-assisted Auger recombination in MBE grown InGaN quantum wells by electron emission spectroscopy. Appl. Phys. Lett. 2020, 116, 091102 10.1063/1.5125605.

[ref56] Johnson J. M.; Chen Z.; Varley J. B.; Jackson C. M.; Farzana E.; Zhang Z.; Arehart A. R.; Huang H. L.; Genc A.; Ringel S. A.; Van De Walle C. G.; Muller D. A.; Hwang J. Unusual formation of point-defect complexes in the ultrawide-band-gap semiconductor β-Ga_2_O_3_. Phys. Rev. X 2019, 9, 041027 10.1103/PhysRevX.9.041027.

[ref57] Saha M. N. Ionization in the solar chromosphere. London, Edinburgh, and Dublin Philosophical Magazine and Journal of Science 1920, 40, 472–488.

[ref58] Philbin J. P.; Rabani E. Electron-hole correlations govern Auger recombination in nanostructures. Nano Lett. 2018, 18, 7889–7895. 10.1021/acs.nanolett.8b03715. 30403875

[ref59] Brendel M.; Kruse A.; Jönen H.; Hoffmann L.; Bremers H.; Rossow U.; Hangleiter A. Auger recombination in GaInN/GaN quantum well laser structures. Appl. Phys. Lett. 2011, 99, 031106 10.1063/1.3614557.

[ref60] Chou H. H.; Wong G. K. Experimental determination of the density dependence of electron-hole correlation in electron-hole liquid. Phys. Rev. Lett. 1978, 41, 1677 10.1103/PhysRevLett.41.1677.

[ref61] Grivickas V.; Ščajev P.; Bikbajevas V.; Korolik O. V.; Mazanik A. V. Carrier dynamics in highly-excited TlInS_2_: Evidence of 2D electron-hole charge separation at parallel layers. Phys. Chem. Chem. Phys. 2019, 21, 2102–2114. 10.1039/C8CP06209A. 30640336

[ref62] Ščajev P.; Soriu̅tė V.; Kreiza G.; Nargelas S.; Dobrovolskas D.; Malinauskas T.; Subačius L.; Onufrijevs P.; Varnagiris S.; Cheng H. H. Temperature and spatial dependence of carrier lifetime and luminescence intensity in Ge_0.95_Sn_0.05_ layer. Mater. Sci. Eng., B 2021, 270, 115204 10.1016/j.mseb.2021.115204.xy3.

