# Supplementary material for: Self-Trapped-Exciton Radiative Recombination in β–Ga2O3: Impact of Two Concurrent Nonradiative Auger Processes
Source: ACS Appl Electron Mater. 2025 Feb 17;7(5):1829–41. doi: 10.1021/acsaelm.4c02099 (PMC11905285; doi:10.1021/acsaelm.4c02099)
Supplement: Supplementary file 1 — el4c02099_si_001.pdf [file el4c02099_si_001.pdf]

Supporting Information for

Self-trapped-exciton radiative recombination in β–Ga_2O_3: Impact of two concurrent non-radiative Auger processes

Vytautas Grivickas[a]*, Patrik Ščajev[a], Saulius Miasojedovas[a], Lars Voss[b], Paulius Grivickas[c]

[a] *Institute of Photonics and Nanotechnology, Faculty of Physics, Vilnius University, Saulėtekio av. 3, 10257, Vilnius, Lithuania*
[b] *Materials Engineering Division, Lawrence Livermore National Laboratory, 7000 East Ave, Livermore, CA 94550, United States*
[c] *Lawrence Livermore National Laboratory, 7000 East Ave, Livermore, CA 94550, United States*

* Email: vytautas.grivickas@ff.vu.lt

In this Supplementary Material we present additional results obtained in β–Ga_2O_3 samples. In section S1 - the Raman scattering measurements, in S2 - the optical transmission spectra at the absorption edge under different polarization, in S3 – CL unpolarized spectra, in S4-S5 - PL unpolarized mapping after confocal CW excitation with strong excitation below the bandgap by 355 nm light. In S6 - data of time-resolved fast PL transients in samples of different plane, in S7 – IQE measurements by integrated PL in an integrating sphere, in S8 – expression for calculating the average excited density.

Fig. S1 shows the Raman spectra obtained from (-201) and (001) oriented β–Ga_2O_3 substrates under (//) polarization. The Raman lines in the region of 100-200 cm^{-1} primarily correspond to the translation and rotation of the tetrahedral and octahedral chains. The figure indicates that the Raman lines in plane (-201) and in (001) planes are nearly identical under polarization E//b. In the (001) plane, the intensity distribution relative to the polarization angle is symmetric about the E//*b* and E//*a* axes.

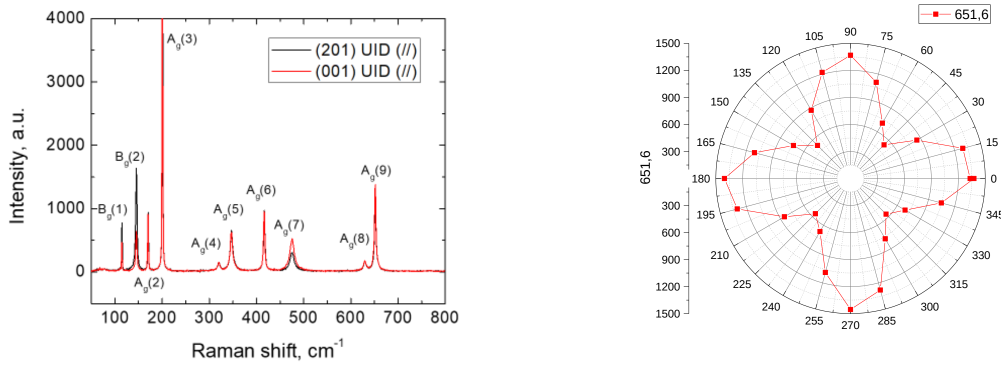

Figure S1. (left) Raman spectra in the UID samples after confocal 532 nm excitation. (right) Distribution of polarization intensity for the optical phonon $A_g(9)$ mode at 651.6 cm^{-1} line in the (001) UID Sn-doped sample.

The Fig. S2 shows optical transmission data at the band edge in UID β–Ga_2O_3 samples. To extract the absorption coefficient at different polarization angles, a constant reflectivity of R = 0.16 was used in the formula for multiple reflectivity [1]. This approach is sufficiently accurate for higher absorption at wavelengths 263 nm or 266 nm. However, it is less reliable for lower absorption, which affects the extraction of the upper band gap at Γ_1^--Γ_1^+ transitions,

as shown in Fig. 2c. (Obtaining the actual spectral distribution of reflectivity versus polarization angle is a relatively challenging task [2].) In the long-wavelength range, such as that presented in Fig. 1 of the main text, where optical anisotropy is weak, we used dispersion data for the average refractive index in β−Ga_2O_3 substrates and films based on the referenced data [3].

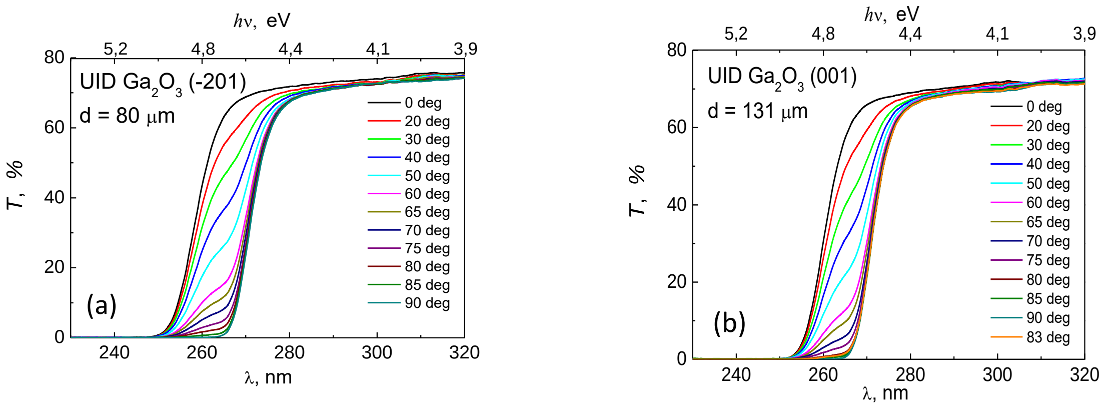

Figure S2. Optical transmission around the band-edge spectral range in polished thinned UID β-Ga_2O_3 samples of (a) in (-201) and (b) in (001) plane. Angle of linear polarization is indicated as described in the main text.

Fig. S3 summarizes the CL spectra obtained after constant defocused e-beam excitation with energy well above the bandgap, as described in the main text (Methods). The figure reveals a significant regression in emission intensity observed in Sn-doped β−Ga_2O_3 samples, similar to the PL spectra presented in the main text (Fig. 3a). The CL band width is ~0.77 eV, with no other emission bands detected across the visible and NIR range. The differences between PL and CL could be partialy attributed to the experimental conditions. For CL, near-surface excitation is performed, continuous-wave (CW) excitation is used, and the collection is carried out with unpolarized light. In contrast, PL excitation involves bulk-like excitation with time-integrated measurements (see Methods). The surface excitation in CL can be affected by emitting places of very different defects (see S4 below). Additionally, surface excitation in CL may be influenced by emissions originating from various defect sites (see S4 below).

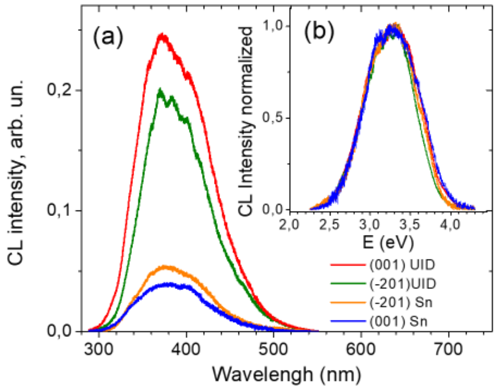

Figure S3. Unpolarized CL spectra in β-Ga_2O_3 samples from (-201) and (001) planes: **(a)** *vs* wavelength in linear scale **(b)** The same spectra normalized to emission peak at 3.27 eV *vs* quantum energy. The band width is 0.77±0.05 eV.

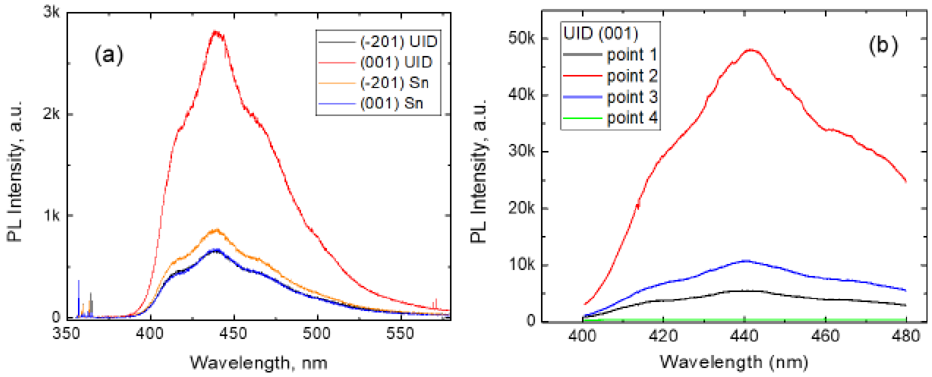

Figure S4. Unpolarized PL intensity obtained by 355 nm CW light of confocal beam focused to a spot of ~2 μm diameter at the surface of β-Ga_2O_3 samples vs wavelength at RT. The light penetration depth is ~ 2 μm, the light power is 2 mW, and the acquisition time is 5 sec. **(a)** Results from an arbitrary located points in four different samples. The spectra in (-201) UID and (001) Sn sample are overlapped. The sharp lines/replicas near 355 nm are related to Raman scattering lines. **(b)** PL from arbitrary selected four points in the (001) UID sample.

S4. To provide extended PL mapping of β-Ga_2O_3 samples, we used confocal CW excitation with 355 nm light (within the exciton band) and focused it strongly to a 2 μm diameter spot projected directly onto the sample surface. The obtained results are shown in **Fig. S4** and the panels of **Fig. S5**. DAP spectra were observed at particular spots, with similar spectral results reported in earlier studies, such as those by Wang et al. (see Ref. [38] in the main text).

The DAP-shaped features may correspond to cases where the sample contains a sufficient density of compensating Ga vacancies, and the excitation light facilitates vacancy recharging through TPA. This transition can be supported either by trap-assisted subgap absorption or by TPA band-to-band excitation, the latter of which was reported by Wang et al. [38]. Another possibility is that vacancies or compensating impurities are located at surface defects, possibly due to the slicing properties of β-Ga_2O_3 samples. This scenario was reported by Huso et al. (see Ref. [39] in the main text), where CW excitation at 3.5 eV was generated using a Xe lamp source.

Our investigation confirms the presence of DAP processes in our samples, consistent with Ref. [38], although such occurrences are very rare.

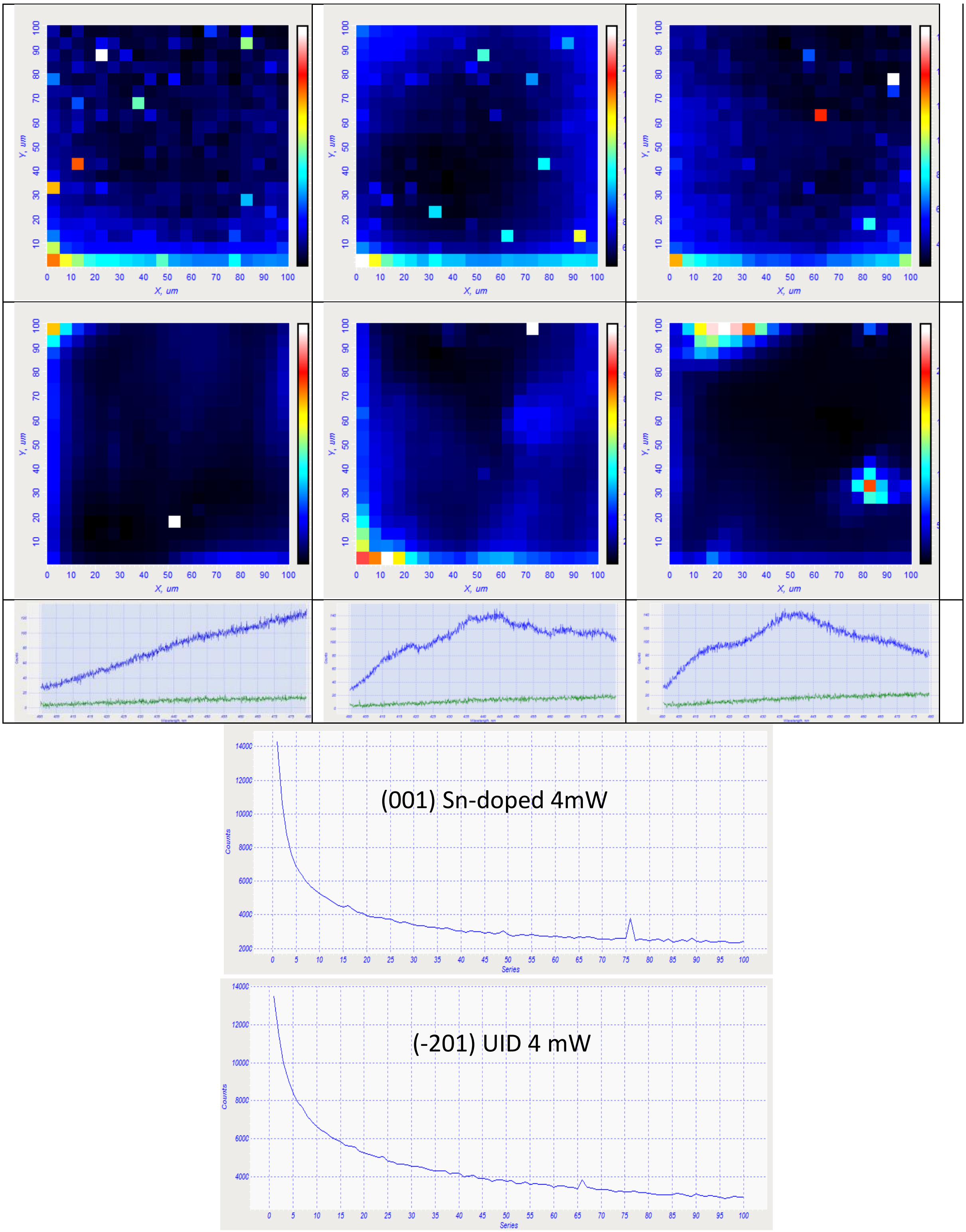

Figure S5. (top panels) PL intensity mapping from 100x100 μm^2 area in two β-Ga_2O_3 samples measured using focused CW 355 nm light with 4 mW power and 1 sec acquisition time. (top row images) (001) Sn-doped sample. (middle row images) the (001) UID sample and corresponding spectra of a bright and a dark spot. (bottom panels) PL intensity degradation in the bright spot during 100 series of the 1 sec acquisition time with 4 mW light power in two β-Ga_2O_3 samples.

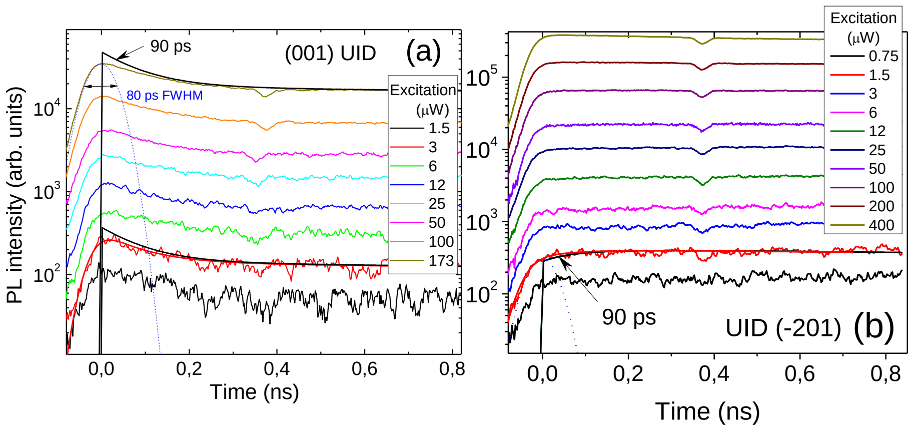

Figure S6. The initial stage (t < 1 ns) of PL decays (a) in the (001) and (b) in the (-201) plain UID β-Ga_2O_3 samples. The excitation is produced by 200 fs laser pulse. The rise of PL is determined by convolution of Gauss excitation pulse and the system temporal resolution (80 ps). A fit to PL is indicated by 90 ps initial transition. The PL intensity rises in (-201) plane while it decreases in (001) plane. This feature does not change as a function of excitation power (not normalized to diameter of the excitation spot). In the used excitation range, the recombination time is sufficiently longer than 1 ns.

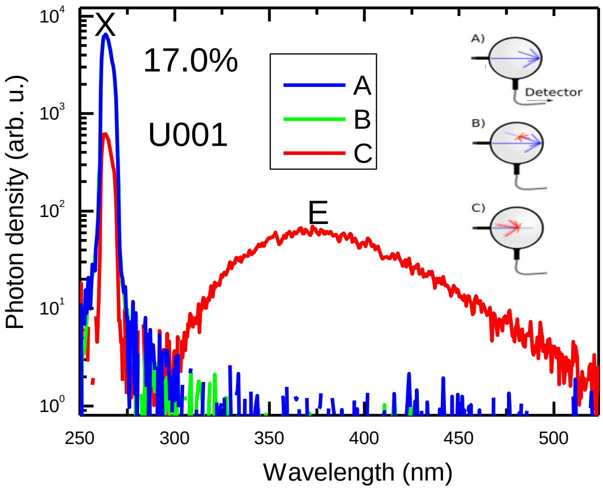

Figure S7. Integrated IQE measurements in UID (001) sample. The residual excitation and emission light is measured under three schemes: A (without sample), B (indirect sample illumination) and C (direct illumination) of the sample. Sample size is < 1 mm^2.

S7. For the IQE determination, the measurements were performed in three configurations: A (empty sphere), B (indirect illumination of the sample), and in C (direct illumination), as depicted in Fig. S7, on small size samples placed in a sphere coated with ultra-white $BaSO_4$ and with SMA port connected detector to a waveguide spectrometer. The waveguide spectrometer measured spectral intensities, which were then converted to photon density at specific wavelengths, taking into account the system's spectral sensitivity correction function. The obtained spectrum consists of two parts: the residual laser excitation light, denoted as X, and emitted light, denoted as E. Both components were integrated separately,

with subscripts indicating the respective measurement. Finally, the sample absorption A and the IQE were calculated as $A = 1 – X_C/X_B$, $\eta = 100\%\cdot(E_C – (1 – A)\cdot E_B)/(A\cdot X_A)$ [4]. In the B configuration, the spectra are very similar to those from A due to the small size of the samples used.

S8. The excited exciton density was calculated using the absorption coefficient α. The generated electron-hole density exhibits an exponential in-depth distribution given by $\Delta n(z) = \Delta N_0 \exp(–\alpha z)$, where $\Delta N_0 =(1–R)\, \alpha\Phi/h\nu$ represents the density near the surface. Here, R = 0.16 is the reflection coefficient, Φ is the excitation fluence, $h\nu$ is the pump photon energy. Near the sample surface, the nonlinear recombination effect is significantly stronger than at greater depths, necessitating the calculation of an average in-depth carrier density. The averaged in-deph carrier density is calculated according to Ref. [5] using the equation $\Delta n = 0.5\Delta N_0(1+\exp(–\alpha d))$. Under strong absorption conditions, this equation simplifies to $\Delta n = 0.5\Delta N_0$.

References

1. Pankove, J. I. „Optical processes in semiconductors“. Dover Publications, Inc. NY 1971.
2. Adnan, Md. M. R.; Verma, D.; Sturm, C.; Schubert, M.; Myers, R. C. Is the Beer-Lambert law in β-Ga2O3 applicable? Spectral and polarization dependent absorption and photoresponsivity. Preprint. **2023**, https://www.researchgate.net/publication/371175535
3. Onuma, T.; Saito, S.; Sasaki, K.; Masui, T.; Yamaguchi, T.; Honda, T.; Kuramata, A.; Higashiwaki, M. Spectroscopic ellipsometry studies on β-Ga2O3 films and single crystal. *Jap. J. of Applied Physics* **2016**, 55, 1202B2, DOI: 10.7567/JJAP.55.1202B2
4. https://DOI.org/10.1038/s41598-019-51718-4
5. Klein, P. B.; Myers-Ward, R.; Lew, K.-K.; VanMil, B. L.; Eddy, Jr. C. R.; Gaskill, D. K.; Shrivastava, A.; Sudarshan, T. S. Recombination processes controlling the carrier lifetime in n^- 4H–SiC epilayers with low $Z_{1/2}$ concentrations, J. Appl. Phys. 2010, 108, 033713.
